# Force-induced ion generation in zwitterionic hydrogels for a sensitive silent-speech sensor

**DOI:** 10.1038/s41467-023-35893-7

**Published:** 2023-01-13

**Authors:** Sijia Xu, Jie-Xiang Yu, Hongshuang Guo, Shu Tian, You Long, Jing Yang, Lei Zhang

**Affiliations:** 1 Department of Biochemical Engineering, School of Chemical Engineering and Technology, Frontier Science Center for Synthetic Biology and Key Laboratory of Systems Bioengineering (MOE), Tianjin University, Tianjin, 300350 China; 2 Institute of Theoretical and Applied Physics, School of Physical Science and Technology, Soochow University, Suzhou, 215006 China

**Keywords:** Gels and hydrogels, Materials for devices

## Abstract

Human-sensitive mechanosensation depends on ionic currents controlled by skin mechanoreceptors. Inspired by the sensory behavior of skin, we investigate zwitterionic hydrogels that generate ions under an applied force in a mobile-ion-free system. Within this system, water dissociates as the distance between zwitterions reduces under an applied pressure. Meanwhile, zwitterionic segments can provide migration channels for the generated ions, significantly facilitating ion transport. These combined effects endow a mobile-ion-free zwitterionic skin sensor with sensitive transduction of pressure into ionic currents, achieving a sensitivity up to five times that of nonionic hydrogels. The signal response time, which relies on the crosslinking degree of the zwitterionic hydrogel, was ~38 ms, comparable to that of natural skin. The skin sensor was incorporated into a universal throat-worn silent-speech recognition system that transforms the tiny signals of laryngeal mechanical vibrations into silent speech.

## Abstract (web summary)

The development of sensitive hydrogel sensor without additional mobile ionic species is challenging. Here, the authors demonstrate force-induced ion generation in a skin sensor based on zwitterionic hydrogel, and build a throat-worn silent-speech recognition system with high recognition accuracy.

## Introduction

**Fig. 1:**
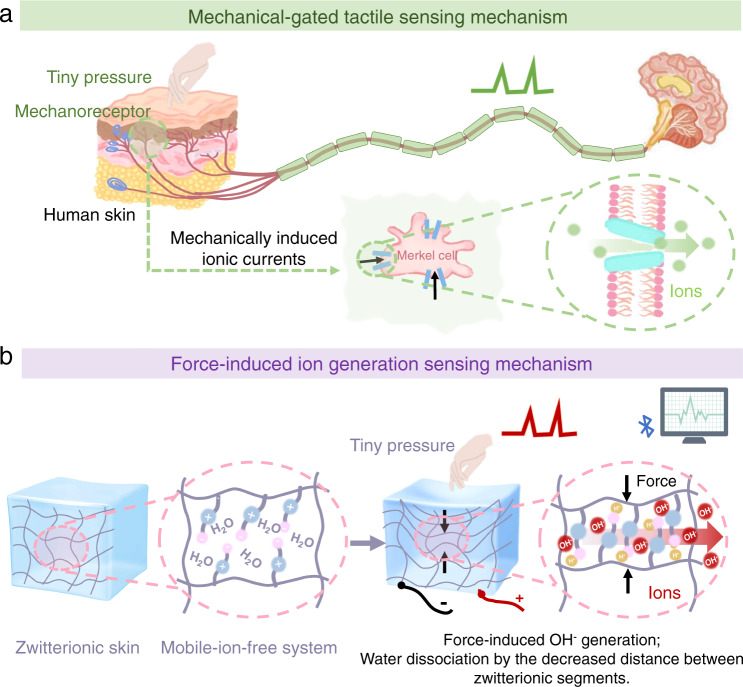
Schematics of (a) the mechanically gated tactile-sensing mechanism of human skin and (b) force-induced ion-generation sensing mechanism of the zwitterionic hydrogel. In **a**, the mechanoreceptors on human skin respond to tiny pressures and mechanically gated ion channels (Piezo2 protein) open to allow transmembrane influx of ions. In **b**, the zwitterionic PCBMA hydrogel deforms under a tiny pressure and generates ions in a pure water system with no additional mobile ions. The deformation decreases the distance between the zwitterions on the polymer backbones, dissociating water into mobile OH^−^. The instantaneous increase in ionic concentration produces ionic currents that improve the sensing sensitivity of the hydrogel.

The human skin can sensitively detect tiny pressures such as the impressions of breezes and mosquito bites^1^. Soft mechanoreceptors in the skin transduce mechanical forces into electrophysiological signals through an ionic conduction mechanism. Mechanically gated ion channels (e.g., Piezo2 protein) on these mechanoreceptors (e.g., Merkel cells) open in response to an external pressure, inducing transmembrane movements of ions^2–4^. The consequent ionic currents are interpreted by the human brain (Fig. 1a).

Recent skin-like sensors such as electronic and ionic skins have overcome the device-to-tissue mechanical mismatch that hinders conventional electronic equipment^5–7^. Compared to electronic skins^1,8–10^, ionic skin enables the development of more transparent, biocompatible, and skin-identical ionic conduction-based sensors from hydrogel^5,11–18^. However, developing an intrinsically sensitive hydrogel sensor without additional mobile ionic species (e.g., inorganic salts^11,15,19^, electrolyte salts^20^, or ionic liquids^13,21,22^) is rendered challenging by the ambiguous relationship between the structure and pressure-response sensitivity of a material.

Inspired by the sensory behavior of skin, we proposed that a tiny pressure can greatly and instantaneously increase the number of ions; thus, strengthening the ionic current in a hydrogel. This mechanism is crucial for developing an intrinsically sensitive skin-like hydrogel sensor. Here we investigate the detection sensitivities of two categories of monomer-based hydrogels (zwitterionic and nonionic hydrogels) in a pure water environment with no mobile-ion additives. We reveal the zwitterionic poly (carboxybetaine methacrylate) (PCBMA) hydrogels that generate ions under an applied force in a mobile-ion-free system. In this system, water dissociates as the distance between zwitterions decreases under pressure (Fig. 1b). Interestingly, this phenomenon was absent in the zwitterionic sulfobetaine methacrylate (PSBMA) hydrogel. Furthermore, zwitterionic segments in which cationic and anionic charges are immobilized on the same molecule provide migration channels for the generated ions, significantly facilitating ion transport^23^. Under these combined effects, the ionic currents are instantaneously increased under a tiny pressure, indicating the usefulness of PCBMA hydrogel as a sensitive skin sensor.

Based on the sensitive PCBMA skin sensor, we establish a throat-worn speech recognition system (TW-SSRS) for silent speech interface (SSI), which can achieve unspoken and hidden communication between users, or user and computer, without any voice and identifiable action^24,25^. Instead of the biological electronic signal recognition during subvocalization or thoughts in current advanced SSI technologies, e.g., surface electromyography^26,27^ and electro-encephalographic^28,29^, the TW-SSRS utilizes the tiny signals of laryngeal mechanical vibrations that were more controllable and possessed a higher signal-to-noise ratio, thus exhibiting a superior recognition accuracy (95%), universality, more privacy, and motion suitability.

## Results

### Unique properties of force-induced ion generation in the PCBMA hydrogel

All recent studies consider that mobile-ion additives are necessary for ionic conduction and pressure response in hydrogel-based skin^30^. Force-induced deformation decreases the cross-sectional area of a hydrogel, preventing the mobility of ions that influences the electronic signal^31^. Therefore, it has been regarded that the pressure-response sensitivity of a hydrogel highly relies on its initial mobile-ion concentration. Here, we reveal that the mechanical response sensitivity of a hydrogel depends not on its mobile-ion additives but on the intrinsically chemical structure of the hydrogel.

**Fig. 2:**
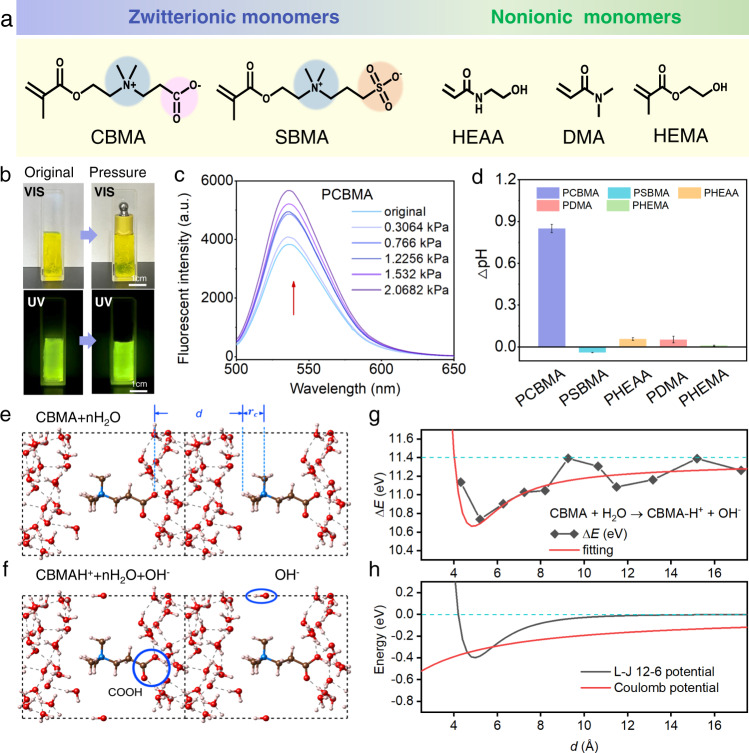
Force-induced ion generation in the hydrogels. **a** Two families of hydrogel monomers (zwitterionic CBMA and SBMA and the nonionic HEAA, DMA, and HEMA) are linked by MBAA, forming hydrogels in a mobile-ion-free system. **b** Photographs of the PCBMA hydrogel before and after an external force application under visible light (upper) and ultraviolet light (lower). **c** Fluorescence emission spectra of PCBMA hydrogel under various stresses (excitation wavelength = 490 nm). a.u. arbitrary unit. **d** pH change in hydrogels under a stress of 2.07 kPa. The hydrogels are sized 15 mm × 10 mm × 10 mm. Data are reported as their means ± SDs (*N* ≥ 3). **e** Density functional theory results of the PCBMA hydrogel. The periodic unit cell (enclosed in the dashed-edged box) contains one CBMA molecule and 40 water molecules. The spaces between neighboring CBMAs are also filled with 40 water molecules. The blue, red, brown, and white colors represent the N, O, C, and H atoms, respectively. The definitions of $d$ and ${{{\mbox{r}}}}_{{{\mbox{c}}}}$ are also displayed. **f** The formation of COOH and one mobile OH^−^ ensure a neutral charge in this system. **g** Plots of ΔE(*d*) versus distance *d* between N(CH_3_)^3+^ and COO^−^ on neighboring CBMA segments. The red solid line is fitted to Eq. (2) in the main text. **h** Plots of the L–J 12-6 and Coulomb terms versus *d* (black and red solid lines, respectively). The dashed line represents the energy at infinite *d*.

To better understand the structure–performance relationship of hydrogel sensor sensitivity, we designed and explored two families of pristine homogeneous hydrogels: zwitterionic hydrogels (PCBMA and PSBMA) and nonionic hydrogels [poly (N-(2-hydroxyethyl) acrylamide (PHEAA), poly (N, N-dimethylacrylamide) (PDMA), and poly (2-hydroxyethyl methacrylate) (PHEMA)]. These monomers and synthetic route of hydrogels are shown in Fig. 2a and Supplementary Fig. 1, respectively, and their chemical structures are verified in Supplementary Fig. 2. Within these pure water systems with no additional mobile ions, only the concentrations of the existing trace H^+^ or OH^−^ ionized by water molecules in the hydrogels can change, and the pH of them were all neutral (Supplementary Fig. 3). To investigate the transient variations of the H^+^/OH^−^ concentrations in the hydrogels induced by external forces, we applied the pH-indicator dye 2,7-bis(carboxyethy1) carboxyfluorescein (BCECF) (Supplementary Fig. 4), which exhibits pH-dependent absorption maxima at 490 and 450 nm^32^. When a series of insulative weights were placed on the hydrogels, the fluorescent color of PCBMA transformed from dark- to light-green under irradiation (Fig. 2b). The fluorescence emission spectrum of PCBMA was obviously enhanced as the stress increased from 0 to 2.07 kPa (Fig. 2c). The pH of PCBMA increased by ~0.85 under the maximum stress (2.07 kPa), whereas the pH values of the other hydrogels (including zwitterionic PSBMA) negligibly responded to stress (Fig. 2d and Supplementary Fig. 5). Surprisingly, an external force increased the mobile OH^−^ generation in PCBMA by ~7.1 times, but generated neither mobile H^+^ nor OH^−^ ions in the nonionic and PSBMA hydrogels.

To understand the mechanisms of force-induced OH^-^ generation in the PCBMA hydrogels, we investigated the behaviors of the zwitterionic segments as they approached each other during compressive deformation of the polymeric networks. For this purpose, density functional theory (DFT) calculations were employed. As presented in Fig. 2e–h and Supplementary Note 1, the equilibrium reaction CBMA + H_2_O = CBMAH^+^ + OH^−^ occurred in the hydrogel system. The energy variation ΔE(*d*) in the system was calculated before and after water dissociation as follows:

1 $$\Delta {{{{{\rm{E}}}}}}(d)={{{{{\rm{E}}}}}}_{{{{{{\rm{CBMAH}}}}}}^{+}+{{{{{\rm{nH}}}}}}_{2}{{{{{{\rm{O}}}}}}+{{{{{\rm{OH}}}}}}}^{-}}(d)-{{{{{\rm{E}}}}}}_{{{{{{\rm{CBMA}}}}}}+{{{{{\rm{nH}}}}}}_{2}{{{{{\rm{O}}}}}}}(d)-{\mu }_{{{{{\rm{H}}}}}_{2}O},$$

where $d$ is the nearest distance between the H atom in the cationic N(CH_3_)^3+^ group and the O atom of the anionic COO^−^ group on the neighboring CBMA segments, and ${\mu }_{{{{{\rm{H}}}}}_{2}O}$ is the chemical potential of a single water molecule.

As shown in Fig. 2g, ΔE(*d*) decreased as $d$ reduced from infinity to 5.0 Å. This result indicates that as the cationic and anionic motifs of neighboring CBMA segments approach each other, the dissociation of water molecules into OH^−^ requires lower energy. The fluctuations in ΔE(*d*) around 5–6 Å (corresponding to the size of water molecules) are attributable to thermal dynamics. The ΔE(*d*) curve was approximately fitted to a combination of the Van der Waals (vdW) potential under Lennard–Jones (L–J) 12-6 formation and the Coulomb potential:

2 $$\Delta {{{{{\rm{E}}}}}}(d)=\Delta {{{{{\rm{E}}}}}}_{0}+\varepsilon \left[{\left(\frac{{r}_{0}+{r}_{c}}{d+{r}_{c}}\right)}^{12}-{\left(\frac{{r}_{0}+{r}_{c}}{d+{r}_{c}}\right)}^{6}\right]-\alpha {k}_{e}\frac{{{{{{\rm{e}}}}}}^{2}}{d+{r}_{c}},$$

where ΔE_0_ is ΔE(*d*) at infinite distance, *ε* is the coefficient of the L–J 12-6 energy, ${r}_{0}$ corresponds to the distance for zero potential energy of the L–J 12-6 energy, ${r}_{{{\mbox{c}}}}$ is the size parameter of the cationic N(CH_3_)^3+^ groups, $\alpha$ is the effective screening parameter of the Coulomb potential, ${k}_{e}$ is the Coulomb constant, and ${{\mbox{e}}}$ is the elementary charge.

According to the fitting curve, ΔE(*d*) decreases by ~0.66 eV (15 kcal/mol) as the cationic and anionic groups on neighboring CBMAs approach from infinity to 5.0 Å under external forces. Accordingly, the equilibrium of CBMA + H_2_O = CBMAH^+^ + OH^−^ shifts toward the right side of the equation and the generated mobile OH^−^ ions increase the pH of the system, consistent with the experimental results. To understand the cause of the distance-dependent ΔE(*d*), the van der Waals potential curve was separately fitted to the Lennard–Jones (L–J) 12-6 and Coulomb potential (Fig. 2h). The van der Waals potential (L–J 12-6 energy) mainly contributed to the balance distance at ~5.0 Å, whereas the Coulomb potential with respect to $d$ was a monotonically increasing curve. Under an external force, the polymer network deformed and $d$ decreased to below 10 Å. During this process, the van der Waals potential remained almost constant at near-zero, while the Coulomb potential dominated the increasing total energy. That is, as $d$ decreased, the H^+^ ions were forced toward the anionic COO^−^ groups via strong repulsive interactions with nearby N(CH_3_)^3+^, allowing easy capture of H^+^ by COO^−^. Owing to the zwitterionic groups, the Coulomb potential mainly depended on the distance-dependent ΔE(*d*). Such Coulomb interactions explain the force-induced OH^−^ generation in the PCBMA hydrogel (see Supplementary Fig. 6). In the nonionic hydrogels, where Coulomb interactions between the neighboring segments were absent, no mobile ions were generated.

Interestingly, the DFT results also revealed why no ions were generated in the zwitterionic PSBMA hydrogel (Supplementary Fig. 7a, b). Under strong self-associative interactions^33^, the smallest distance between neighboring SMBA segments was $d$ = 2–3 Å, akin to hydrogen-bond distances with no space for water molecules (see Supplementary Fig. 7c). Clearly, the polymer network deformation under an external pressure could not break the hydrogen bonds so $d$ was always approximately balanced. Therefore, ΔE(*d*) remained too constant for mobile OH^−^ generation.

**Fig. 3:**
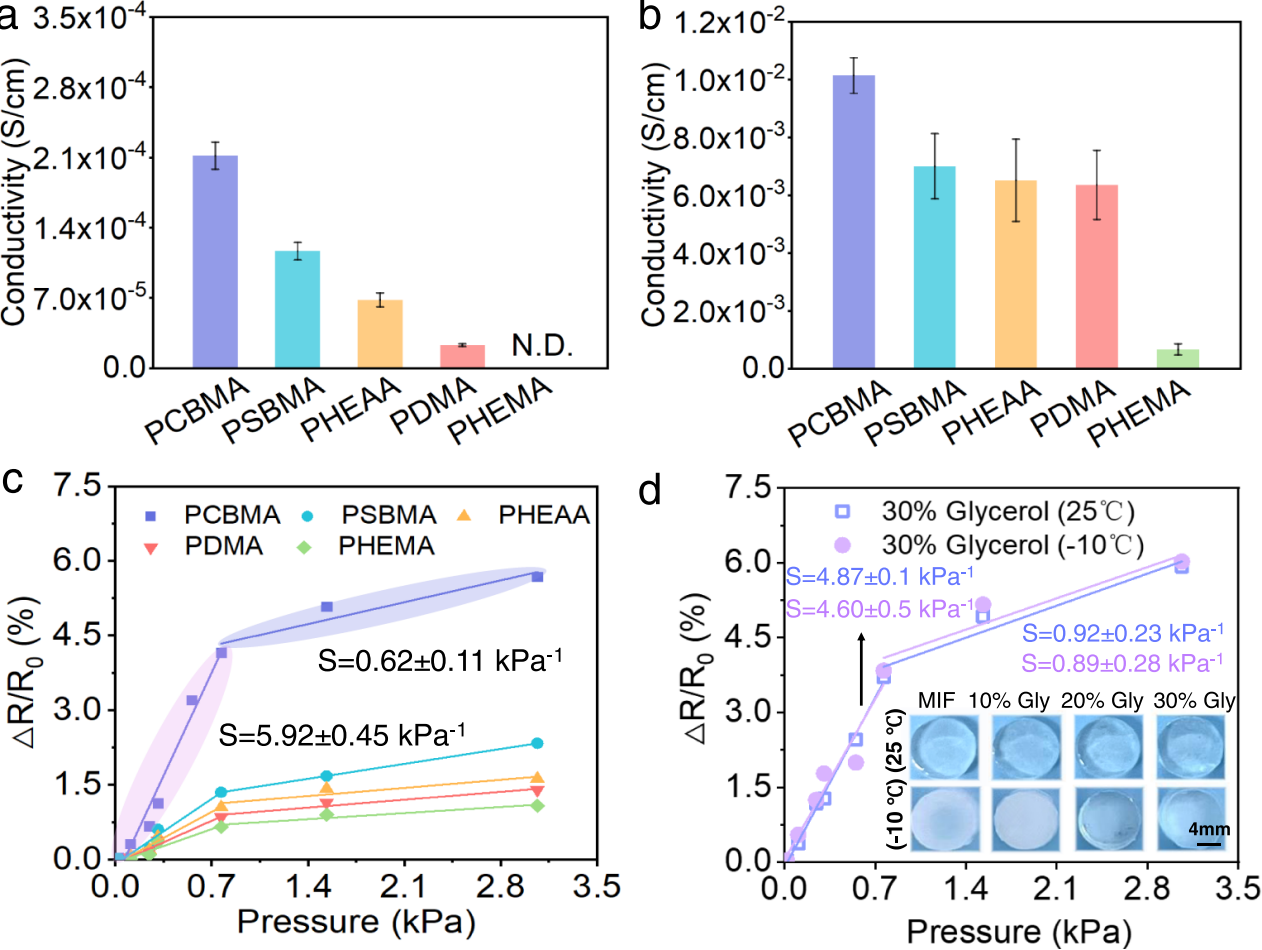
Conductivity and Sensitivity of the hydrogels. **a** Ionic conductivities of the hydrogels in the mobile-ion-free and **b** mobile-ion-added systems, respectively. Data are reported as their means ± SDs (*N* ≥ 3). N.D. not detected. **c** Comparison of hydrogel sensitivities. The correlation coefficients of the high-sensitivity and low-sensitivity phases of the PCBMA hydrogel are *R*^2^ = 0.968 and *R*^2^ = 0.906, respectively. **d** Photos and sensitivity curves of the cryoprotectant-based anti-freezing PCBMA hydrogels at 25°C and −10°C. The correlation coefficients of the high-sensitivity and low-sensitivity phases of the PCBMA hydrogels at 25°C (or −10°C) are *R*^2^ = 0.994 (or 0.925) and *R*^2^ = 0.970 (or 0.952), respectively.

Under an applied voltage, the mobile ions pass through the polymeric network, forming the ionic currents that are fundamental to sensing skin-like materials. Therefore, we measured the conductivities of the zwitterionic and nonionic hydrogels as a proxy of ion mobility. In both the mobile-ion-free and ion-added systems (Fig. 3a, b), the zwitterionic hydrogels were more conductive than any of the nonionic hydrogels, and PCBMA exhibited a higher conductivity than the other hydrogels. It was inferred that the zwitterionic segments in the hydrogels provide a migration channel that facilitates ion transport, consistent with previous reports^34–36^.

### Sensing sensitivity of hydrogels

The pressure-response sensitivities of the hydrogels were investigated in a manually fabricated, compact side-by-side electrode configuration (see Supplementary Fig. 8). The hydrogels were fixed in the middle of the hollow PDMS elastomer and placed on the top of two gold electrodes. Within this configuration, the hydrogels are deformed only at the compressed position; thus, providing stable and reliable pressure-response electronic signals. In the mobile-ion-free system, the PCBMA hydrogel exhibited the maximum change in resistance signal under a tiny external force (0.77 kPa). All other hydrogels, including PSBMA, were insensitive to the applied stress (Supplementary Fig. 9). Under tiny stresses (0–0.8 kPa), the sensitivity (S) of the PCBMA hydrogel was 5.92 ± 0.45 kPa^−1^ (five times higher than those of the nonionic hydrogels; S = 1.15 ± 0.29), reducing to 0.62 ± 0.11 kPa^−1^ in the range 0.8–3 kPa (Fig. 3c). The results indicated that under a tiny pressure, the ions generated in PCBMA significantly increased the ionic-current change, providing a more sensitive sensing signal than in the other hydrogels. However, as the stress increased, the number of generated mobile ions no longer increased in proportion, leading to a significant decline in PCBMA sensitivity at high pressures (S = $\frac{\Delta R}{{R}_{0}\cdot \Delta P}$). We further tested the ion generation and sensitivity of PCBMA hydrogels with the decrease of water contents. Owing to the unique force-induce ion generation mechanism, the cryoprotectant-based PCBMA hydrogel still presents satisfactory sensitivity, as well as presenting anti-freezing performance (Fig. 3d and Supplementary Fig. 12). Interestingly, although mobile-ion additives increased the conductivities of all hydrogels (Fig. 3b), they did not increase the sensitivities of the hydrogels even significantly decreased their sensitivities at higher ion concentrations. (Supplementary Fig. 9–11). When the mobile ions are added to the hydrogel system, there would be more elements in the equilibrium influenced by the external stress. For example, as the low-concentrated Na^+^ in systems, force-induced ions (OH^−^ and Na^+^) could be generated or increased to ensure the pressure-response sensitivity, while this phenomenon would be negligible as the high-concentrated Na^+^ addition (Supplementary Fig. 11, Supplementary Note 2). Because forward tendency was completely inhibited in equilibrium, and the inhibition was proportional to the mass and charge numbers of added cations (Cs^+^ and Mg^2+^). These all-above results demonstrated that the sensing sensitivity of hydrogels more relies on the instantaneously force-induced ion increase, which is attributable to the chemical structure of the materials.

Overall, the sensing sensitivity of hydrogels (S = $\frac{\Delta R}{{R}_{0}\cdot \Delta P}$) is directly related to Δ*R*, meaning the internal increase in ionic concentration, either through the force-induced increase in ion number or decrease in hydrogel volume. When the polymer network is deformed, the decreased intermolecular distance can affect the Coulomb and van der Waals interactions between moieties^37^. According to the law of conservation of energy, the intrinsic response sensitivity of hydrogel-based skin sensors is given by:

3 $${{{{{\rm{S}}}}}}\,=\,\frac{\Delta C}{{C}_{0}\cdot \Delta P}=\frac{{{{{{\rm{E}}}}}}_{\Delta p}+{{N}}{{\alpha }}{k}_{e}{{{{{\rm{e}}}}}}^{2}(\frac{1}{d+{r}_{c}}-\frac{1}{{d}_{0}+{r}_{c}})}{({{{{{\rm{E}}}}}}_{0}+{{N}}{{\alpha }}{k}_{e}\frac{{{{{{\rm{e}}}}}}^{2}}{{d}_{0}+{r}_{c}})\cdot \Delta P},$$

where Δ*C* is the increase in ionic concentration contributed by water dissociation, ${C}_{0}$ is the initial ion concentration in the pure water system, Δ*P* is the external pressure, E_Δ*p*_ and ${{{\mbox{E}}}}_{0}$ are the total energy produced under external pressure and the initial total energy, respectively, and $N$ is the volume-unit average number of zwitterionic segments (see Supplementary Note 3 for derivation).

From Eq. (3), we note that: (1) In a nonionic hydrogel, ${{{\mbox{e}}}}^{2}$ approaches zero and S reduces to $\frac{\Delta C}{{C}_{0}\cdot \Delta P}=\frac{\Delta V}{{V}_{0\cdot }\Delta P}$, implying that the sensitivity is related to the force-induced decrease in hydrogel volume (Δ*V*) and the elastic energy of the chains; (2) in the PSBMA hydrogel, *d* approaches *d*_*0*_ and S reduces to $\frac{\Delta V}{{{{{{{\rm{2E}}}}}}}_{0}+2N\alpha {k}_{e}\frac{{{{{{\rm{e}}}}}}^{2}}{{d}_{0}+{r}_{c}}}$, implying that the sensitivity is related to the constant *d*_*0*_ and ${{{\mbox{e}}}}^{2}$, which are determined by the initial crosslinking degree of PSBMA; (3) in the PCBMA hydrogel, the sensitivity depends on the mechanical stimuli, initial *d*_*0*_, and ${{{\mbox{e}}}}^{2}$, owing to the unique property of force-induced ion generation in the polymer network. Moreover, for different applications, the heterogeneous hydrogel based on different types of monomers can be considered. More charged-motif monomers, lower self-association effects among charged motifs, and higher crosslinking degrees can determine the resultant sensitive heterogeneous hydrogels, as well as considering mechanical strength, stretchability, or other required properties.

### Response time of hydrogels

**Fig. 4:**
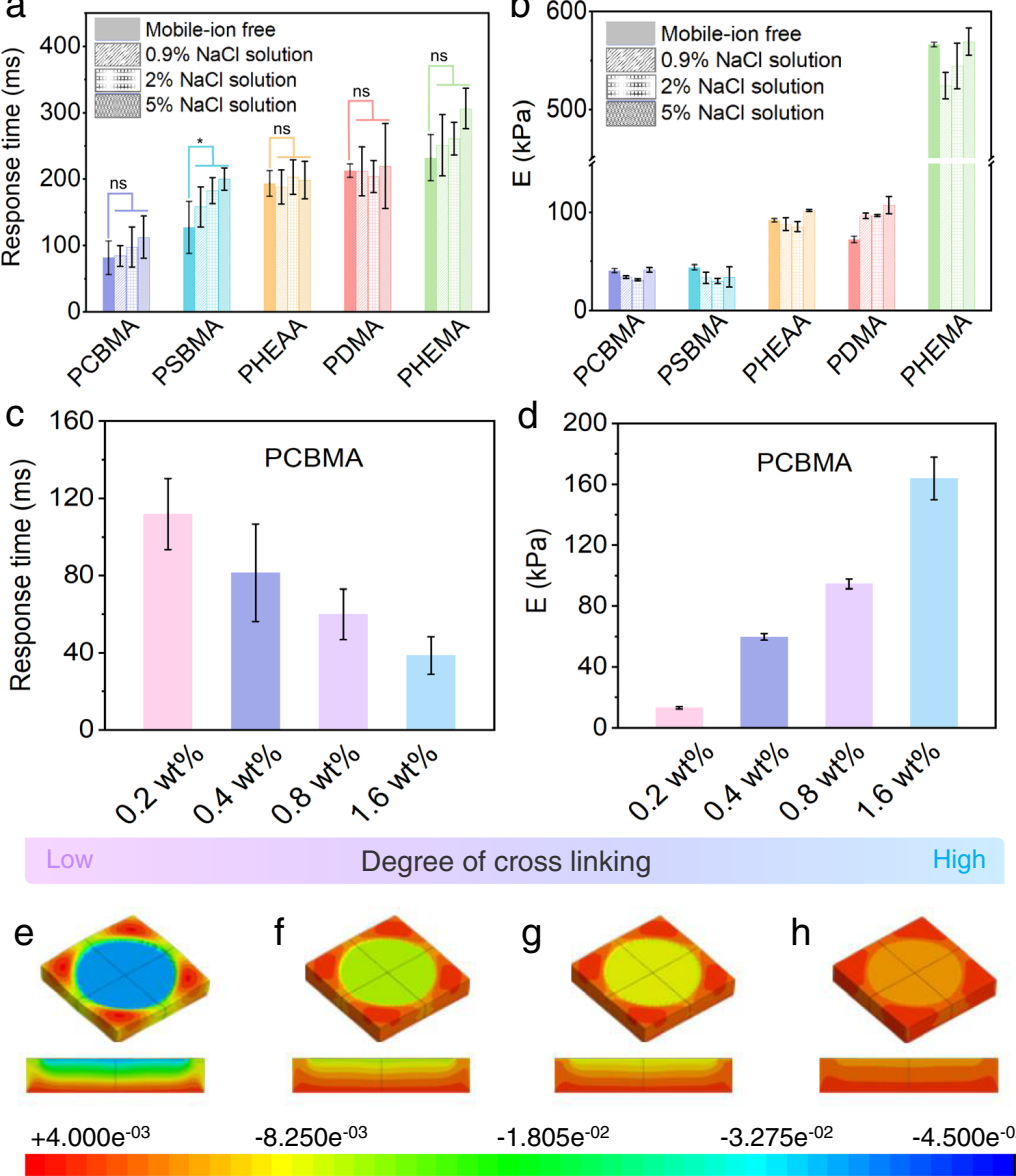
Response time of the hydrogels. **a** Response times of zwitterionic and nonionic hydrogels under a small pressure (0.77 kPa) in the mobile-ion-free and mobile-ion-added (NaCl) systems. Data are reported as their means ± SDs (*N* ≥ 3). ns: no significance. **b** Compression elastic moduli of the hydrogels. Data are reported as their means ± SDs (*N* ≥ 3). **c** Response times and **d**, compression elastic moduli of the PCBMA hydrogels with different crosslinking degrees (cross-linker contents of 0.2 wt%, 0.4 wt%, 0.8 wt%, and 1.6 wt%, left to right) under a small pressure (0.77 kPa). Data are reported as their means ± SDs (*N* ≥ 3). **e**–**h** Displacement of simulation results for PCBMA hydrogels with different crosslinking degree under tiny pressure (0.77 kPa) (the cross-linker contents are 0.2 wt%, 0.4 wt%, 0.8 wt%, and 1.6 wt%, from **e** to **h**). The modulus of hydrogels is assigned according to the data in Supplementary Fig. 16d. The colors of the hydrogels indicate the total displacement.

Another key sensing-performance parameter is the response time (Rt), which determines how quickly the skin sensor reaches its final signal amplitude under an applied pressure. In previous reports, the Rt of hydrogels was considered to increase with elastic modulus because hydrogels are resilient when the pressure is released. Herein, we reveal that Rt is more related to the built-in polymer segments and the crosslinking degree of the network than to the extrinsic elastic modulus. Figure 4a compares the Rts of the monomer-based hydrogels with different polymer segments. In the mobile-ion-free system, the Rts of the zwitterionic PCBMA and PSBMA hydrogels (81.5 and 127.2 ms, respectively) were much faster than those of the nonionic hydrogels (>200 ms). Meanwhile, the compression elastic modulus was lowest in PCBMA (40.8 kPa) and highest (566.3 kPa) in PHEMA (Fig. 4b and Supplementary Fig. 14). However, the Rt of the high-elastic-modulus PHEMA was much slower than that of PCBMA and similar to those of the nonionic PHEAA and PDMA hydrogels. After mobile-ion addition, all hydrogels exhibited an increased Rt but no significant change in elastic modulus. In addition, the Rt of most hydrogels (regardless of the presence or absence of mobile ions) was not significantly influenced by water content and swelling ratio (Fig. 4a and Supplementary Figs. 13 and 15). The exception was the PSBMA hydrogel, in which adding mobile ions increased the swelling ratio and water content via dissociation of self-association interactions between the SBMA segments. These results demonstrated that the elastic modulus, mobile-ion addition, and water content of different monomer-based hydrogels were none of the determining factors of Rt.

To investigate Rt among the same monomer-based hydrogels (Table S1), we prepared PCBMA hydrogels with different crosslinking degrees and verified their crosslinking variations in scanning electron microscopy (SEM) and differential scanning calorimetry (DSC) tests. The hydrogels exhibited negligible differences in their water contents (Supplementary Fig. 16a–c). As shown in Fig. 4c and Supplementary Fig. 17a–d, the Rt of the PCBMA hydrogels was inversely proportional to the crosslinking degree and was exceptionally fast in the highest-crosslinked PCBMA (~38 ms). In fact, the Rt of such highly crosslinked PCBMA was comparable to that of human skin (30–50 ms). The extrinsic elastic modulus, which reflects the intrinsic network density of a hydrogel, was inversely proportional to Rt (Fig. 4d). As expected, finite element method simulations yielded an inverse proportionality between the PCBMA displacement induced by a tiny pressure (0.77 kPa) and crosslinking degree (Fig. 4e–h, Supplementary Fig. 18), consistent with the change in resistance signals (Supplementary Fig. 17). These results can be explained by the low energy dissipation and short time of reaching the stable state in the highly crosslinked hydrogels. That is, the network crosslinking degree plays a key role in different hydrogels with the same monomer.

In summary, Rt is determined by the built-in chemical structure of the polymeric network, including the charged groups on motifs and the crosslinking degree. When developing a rapid-response heterogeneous hydrogel, one must consider the density of the charged segments in the polymeric network. The ionic channels formed by charged motifs can facilitate the transport of ions. The increasing concentrations of ion addition improve the conductivity of hydrogels in Fig. 3a, b, instead of the stress-response sensitivity in Fig. 4a and Supplementary Fig. 9. A rapid-response homogeneous hydrogel requires an appropriate degree of network crosslinking. The high-density charged motifs in highly crosslinked PCBMA not only affect the numbers of generated ions as well as the density of ionic channels in the hydrogel, but also increase the elasticity of the hydrogel; thus, lowering the energy dissipation to reach equilibrium.

### Throat-worn silent-speech sensor

**Fig. 5:**
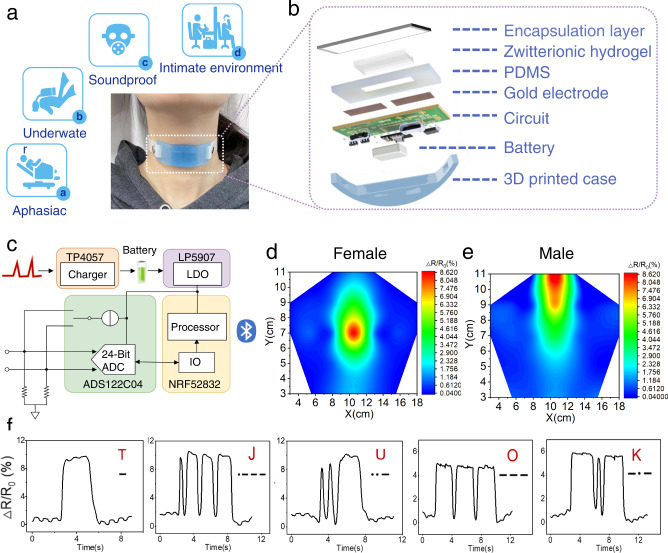
Schematics of the throat-worn silent-speech sensor and real-time speech signals. **a** Specific situations serviced by silent-speech interfaces and a photograph of the throat-worn sensor used by a volunteer. **b** Expanded schematic of the throat-worn sensor showing the zwitterionic sensing hydrogel, hollow PDMS elastomer, wireless printed circuit board, and enclosure architectures. **c** Circuit diagram showing the signal flow in the real-time silent-speech translation system. **d** Resistance signal mappings of the throat vibrations of a female and **e**, male volunteer. There are 27 spatial locations on the throat skin of each volunteer. **f** Recognition of different representative silent-speech signals: “TJU” and “OK”.

Silent-speech interface (SSI) is an advanced technology to achieve unspoken and hidden communication between users or user and computer without any voice and identifiable action^24,25,27,38^. Such systems provide communication services in specific situations when humans cannot produce sound or when voice/visible light transmission is blocked. SSIs assist speech-handicapped patients, privacy information delivery, and communication in underwater environments or noisy and dark conditions (Fig. 5a). However, the currently advanced SSIs commonly suffer from unsatisfactory recognition accuracy, limited motion applicability, and complex training before usage, owing to biological electronic signals that are easily interfered by user’s emotions and exhibit low signal-to-noise ratios.

Therefore, we designed a tiny-pressure-sensitive and quick-response throat-worn skin sensor that captures controllable mechanical vibration signals from the human throat muscle, achieving motion-suitable and precise silent speech (Fig. 5a). The throat-worn sensor consists of a zwitterionic sensing hydrogel, a hollow PDMS elastomer, a wireless printed circuit board (W-PCB), and a three-dimensional (3D)-printed shell (Fig. 5b and Supplementary Fig. 22a, b). Sensing was performed by the mobile-ion-free PCBMA hydrogel, which delivers high sensitivity and a constant response (Supplementary Fig. 19a). Quick detection of dynamic mechanical stimuli (Supplementary Fig. 19b) and durability (Supplementary Fig. 19c) of the PCBMA hydrogel were also confirmed. The outstanding biocompatibility of the hydrogel was verified by its excellent compatibility with both mammalian cell lines in vitro and human skin in vivo (Supplementary Figs. 20 and 21). The W-PCB integrated the multiple functions, including signal conditioning, signal processing, and wireless transmission (Fig. 5c and Supplementary Fig. 22c). The board transmitted the laryngeal mechanical vibration signals into digital signals and then wirelessly transmitted them to a customized signal-recognition system.

To evaluate the optimally sensitive regions of the throat for gender indifference, we selected 27 detection points on the laryngeal skins of male and female subjects (Supplementary Fig. 23). The electrical-resistance signals from the PCBMA sensor were recorded as the mechanical vibrations of human hyoid muscles. Panels d and e of Fig. 5 show the heatmaps obtained by gathering the electrical-resistance results from all target locations via contour mapping in Origin software. The red regions represent the locations of the maximum electrical-resistance signals induced by vibrations of the hyoid muscle. At these locations, the sensitivity and accuracy of signal detection were maximized. The differences in the heatmaps of males and females are attributable to the different laryngeal features of the two genders. Users of the PCBMA-based throat-worn sensor could easily control their tongue movements after simple training; thus, driving the sensor without opening their mouths or uttering any sounds. The communication instruction recognition of the sensor was based on the Morse Code, an international signal-transmission method (Supplementary Fig. 24). The changes in electrical signals caused by short-time and long-time vibrations are represented as dots and dashes, respectively. As shown in Fig. 5f and Supplementary Fig. 24a, the throat-worn sensor could sensitively and preciously recognize all 26 unspoken instructions of the English alphabet. These instructions could be combined into words, phrases, and sentences, greatly expanding the data corpus, and improving the sensor applicability.

### Throat-worn silent-speech recognition system (TW-SSRS)

**Fig. 6:**
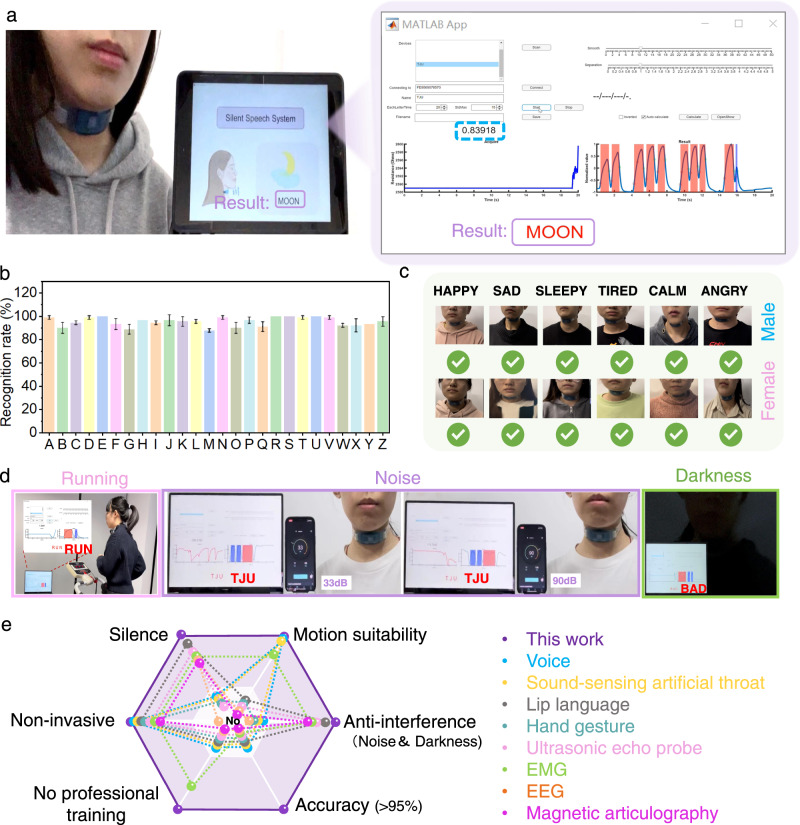
A universal silent speech recognition system (TW-SSRS). **a** User-friendly interface of TW-SSRS for final silent speech expression (left), and an intermediate state in the real-time electrical signal display as silent speech (right). **b** The recognition rates of 26 instructions. Each instruction is repeated for 90 times. **c** Gender indifference test based on six female and male users. **d** Display photos using TW-SSRS during walking and running, under noise and dark environment, respectively. **e** A comparison between this work and previously reported communication technology systems (refs. ^39–46,48,49^).

Finally, the sensitive throat-worn sensor was incorporated into a universal throat-worn silent-speech recognition system (TW-SSRS) that converts the digital signals from the throat-worn sensor into silent speech. The TW-SSRS requires a customized software system (Supplementary Fig. 25). A signal threshold and smoothing functions were set to remove the interference signals, which were probably induced by noises such as swallowing and neck and body movements. The target signals were determined by calculating the maximum and minimum of the signal-peak slope and comparing the time thresholds of the peak width to distinguish between sharp and wide peaks. These processes avoid degradation of the recognition accuracy by baseline drift. The signal conditioning and processing accorded with the established data corpus. Finally, the digital signals were accurately translated into the instructions of silent speech. Figure 6a displays the user-friendly interface (left) and real-time electrical-signal interface (right) of the TW-SSRS. Assisted by the real-time signals (highlighted in blue), users can set the appropriate signal threshold during precommissioning. This functionality improves the availability and versatility of TW-SSRS. As a representative example, the TW-SSRS successfully translated the silent-speech instruction “MOON” (see Supplementary Movie 1).

The current bioelectrical-signal-captured SSI systems have limited recognition accuracy for silent instructions because they are commonly indifferent to users and require to be placed at fixed multiple positions on the users. The unfavorable signal-to-noise ratios in the bioelectrical signals generated by the existing SSI systems (e.g., <−10 dB in EEG) further limit the sensor signal-acquisition locations. As shown in Fig. 5b, the novel TW-SSRS achieved an average accuracy of 95% in the 26-instruction test. To evaluate the universality of TW-SSRS, 12 volunteers (six females and six males) were given six silent instructions related to emotions (“HAPPY,” “SAD,” “SLEEPY,” “TIRED,” “CALM,” and “ANGRY”). The TW-SSRS recognized these words from all volunteers, relying solely on the vibrations of their laryngeal muscles. No complex training processes or voice utterances were required, suggesting the high accuracy and gender indifference of the system (Fig. 6c, Supplementary Movie 2). Notably, the TW-SSRS was motion-suitable for users because mechanical vibration signals with a high signal-to-noise ratio (35 dB) covered a large region of the human hyoid muscles, and this system exhibited anti-interference performance in Supplementary Figs. 26–29. The recognition rates of TW-SSRS during walking (0.8 m/s) and running (1.6 m/s) were maintained as high as 92% (Supplementary Movie 3). The TW-SSRS also performed well in noisy public places and quiet work places, ensuring information accuracy and security (Fig. 6d, Supplementary Movie 4). When the ambient noise reached ~90 dB, the recognition rate of TW-SSRS exceeded 95%, similar to the rate in quiet environments. The performance of TW-SSRS was also unaffected by the visible light intensity (Fig. 6d and Supplementary Fig. 30, Supplementary Movie 5). The operation in silent environments, motion suitability, anti-interference (robustness to noise and darkness), and accuracy (>95%) of the TW-SSRS supersedes those of the current communication systems, including voice recognition^39^, sound-sensing artificial throats^40^, lip-language recognition^41^, hand-gesture recognition^42^, ultrasound-based speech recognition^43^, EMG^26,27,44,45^, EEG^28,45^, and magnetic articulography-based speech recognition^46,47^. Moreover, it requires no professional training and is noninvasive. Fig 6e gives a detailed comparison of TW-SSRS and other communication systems.

## Discussion

In summary, we demonstrated force-induced ion generation in a sensitive skin-mimic sensor based on the zwitterionic PCBMA hydrogel. The sensor was incorporated into a TW-SSRS. The generality of exploiting the intrinsic strain-response sensitivity of hydrogels in a mobile-ion-free system was comprehensively evaluated in experimental tests and DFT calculations. Under a tiny pressure, deformation of the polymer network in the zwitterionic PCBMA hydrogel increased the Coulomb interactions between zwitterions, causing water dissociation and subsequent OH^−^ generation. In addition, the zwitterionic segments provide migration channels for the generated ions. Consequently, the PCBMA skin sensor was five times more sensitive than nonionic hydrogel-based sensors and its response time (~38 ms) was comparable to that of natural skin. The TW-SSRS revealed the superiority of the sensitive PCBMA sensor for tiny mechanical strain, and enabled the silent communication with high recognition accuracy. We believe that the intrinsic sensing sensitivity of hydrogels, based on a general mechano-chemical formula, can offer new insights into the rational design of sensitive strain-response ionic skin sensors for artificial intelligence applications.

## Methods

### Materials

Beta-propiolactone was bought from TCI Development Co., Ltd. 2-(Dimethylamino)ethyl methacrylate (DMAEMA) and Ammonium persulfate (APS) were both obtained from Adamas. SBMA was bought from Sigma-Aldrich Co. HEAA, DMA, and HEMA were all obtained from Heowns. N, N -methylenebis-(acrylamide) (MBAA) and N, N, N, N -tetramethylethylenediamine (TEMED) were bought from Aladdin of Shanghai. Dimethyl sulfoxide (DMSO) and acetone were both obtained from Concord Technology Co., Ltd, Tianjin. Deionized water (18 MΩ cm^−1^) was employed in all experiments.

### Synthesis of CBMA monomer

After removing oxygen by the nitrogen flow, anhydrous acetone (300 mL) and DMAEMA (60 mL) were added to a flask. Stirring at room temperature until they were completely dissolved, beta-propiolactone (21.82 mL, 0.35 mol) was dropwise added to the flask under continuous stirring at 0°C for 2 h. The final product was obtained after washing with ether and then dried under vacuum for 24 h.

### Preparation of the hydrogels

The CBMA (2.1 g, 35 wt%) was dissolved in pure water uniformly. Then MBAA (0.025 g, 0.4 wt%), initiator APS (0.01 g, 0.16 wt%), and TEMED (10 μL) were added into CBMA solution and stirred for 5 min. The solution was transferred into a mold (the thickness is 2.0 mm) and placed at 60°C for 6 h with a one-step radical polymerization to obtain the hydrogels. Eventually, hydrogels were prepared by soaking them in pure water to remove unreacted monomers and initiators and then equilibrated in water for at least 5 days. The water was changed every day. PSBMA, PHEAA, PDMA, and PHEMA hydrogels were prepared with the same process. A series of different crosslinking degrees of PCBMA hydrogels were prepared, as shown in Table S1, Supplementary Information.

### Fluorescence spectrum tests

A Hitachi spectrophotometer (F-2500) was used to measure the fluorescent of the hydrogels. We have confirmed that the ratio of absorption of BCECF at 450 nm (A_450_) and 490 nm (A_490_) linearly changes in the pH range 2–5 (Supplementary Fig. 4). The hydrogels were immersed in a large amount of BCECF solution (2.0 M) for three days prior to use.

### Density functional theory (DFT) simulation

See Supplementary Notes 1 and 2.

### Mechanical strength tests

The mechanical properties were tested via a universal mechanical test machine. A cylindrical sample (diameter = 5 mm, height =2 mm) was used for compression tests. The measurements of compressive modulus were calculated according to the compressive curves.

### Sensing of the hydrogel sensor

An LCR meter (TH2830) was used to measure the resistance change of the hydrogels. The sensitivity, expressed as $\frac{\Delta R}{{R}_{0}\cdot \Delta P}$, is usually used to evaluate the performance of the pressure sensor, where ${R}_{0}$ and $R$ are the initial resistance and resistance under applied pressure, respectively.

### Ionic conductivity assays

All hydrogel samples were equilibrated for 5 days in deionized water or physiological saline (0.9% NaCl) for ionic conductivity assays. The water or saline was changed every day. The hydrogel samples were sandwiched between two electrodes for electrochemical impedance spectroscopy (EIS) measurements using a CHI600E electrochemical workstation. The EIS measurements with a frequency range from 0.01 Hz to 100 kHz were conducted under open-circuit conditions with an excitation voltage of 5 mV. Software ZView (Scribner) was used to fit the impedance data with the Randles equivalent circuit, which contained an electrolyte resistance (*R*_s_) in series with the parallel combination of a constant phase element and a charge transfer resistance (*R*_ct_). The obtained R_s_ values were used to calculate the ionic conductivities of the hydrogel samples using the following equation:

4 $$\sigma=\frac{l}{{R}_{s}\times {{{{{\rm{A}}}}}}},$$

where *σ* is ionic conductivity, A is the cross-sectional area of the sample, and *l* is the thickness of the sample. All experiments were repeated three times.

### Design of wireless data acquisition (DAQ) module

The electronic parts of the wireless DAQ module include an analog front end (ADS122C04, ADI), a microcontroller (nRF52832, Nordic Semiconductor), a battery charge controller (TP4057, UMW), and a voltage regulator (LP5907, TI). The analog front end was used to provide a constant current and measure the voltage for both ends of the electrode. The microcontroller was used to acquire data from the analog front end and calculate the resistance result to wirelessly send via Bluetooth. The battery charge controller was utilized to support a rechargeable 50 mAh lithium-ion polymer battery. The voltage regulator was used to provide working voltage (3.3 V) from the battery to power the electronic part above.

### TW-SSRS performance

The TW-SSRS was built and tested in Windows 10 with Bluetooth connectivity environment. App designer and signal processing toolbox of MATLAB 2019b was used to create GUI (Graphical User Interface) and automatic binning algorithm. In real-time recognition, the sampling frequency was 50 Hz, and the time window length was adjusted by the pause interval of an input signal. In the accuracy test in noisy environment, dark environment, and in motion, five instructions were selected, each instruction was repeated ten times.

The authors affirm that human research participants provided informed consent for the publication of the images in Figs. 5–6, and Movies 1–5.

## Supplementary information

Supplementary Information

Description of Additional Supplementary Files

Supplementary Movie 1

Supplementary Movie 2

Supplementary Movie 3

Supplementary Movie 4

Supplementary Movie 5

## Data Availability

The authors declare that the data that supports the findings of this manuscript can be found in the Supplementary Information and are available free of charge or available from the corresponding author upon request. Source data are provided with this paper.

## Source data

Source Data
