# Supplementary material for: Force-induced ion generation in zwitterionic hydrogels for a sensitive silent-speech sensor
Source: Nat Commun. 2023 Jan 13;14:219. doi: 10.1038/s41467-023-35893-7 (PMC9839672; doi:10.1038/s41467-023-35893-7)
Supplement: Supplementary file 1 — Supplementary Information [file 41467_2023_35893_MOESM1_ESM.pdf]

Supplementary Information for

Force-induced ion generation in zwitterionic hydrogels for a sensitive silent-speech sensor

Xu *et al.*

Supplementary Methods

Water content tests. The hydrogels were immersed in deionized water for different times and weighed for mass M_0. Subsequently, they were dried in a lyophilizer at 25°C for two days and weighed for mass M_1. The water content was calculated using the equation $(M_0–M_1)/M_0$ (%), (S1).

Hydrogel swelling measurements. The swelling ratios of all the samples (a diameter of 5 mm) were determined by calculating the mass ratios of the hydrogel samples before and after swelling. The swelling ratio was calculated using equation M_1/M_0 (%), (S2). Where M_0 is the mass of the hydrogel before swelling equilibrium and M_1 is the mass of the swelling equilibrium hydrogel.

Cytocompatibility tests. The hydrogels and commercial Duoderm dressing were soaked in 500 μL of culture medium for extraction at 37°C for 24 h and the culture medium with no samples was used as the control. Then 3T3 cells (106 cells mL^{-1}) were added into a 48-well plate with different extraction. After 24 and 72 h incubation (37°C, 5% CO_2), the staining reagents were incubated with cells instead of extractions for 15 min and then the cells were observed under a fluorescence microscope.

Characterization. FTIR (ALPHA II, Bruker, Germany) was used to investigate the chemical characteristics of hydrogels. The morphologies of various hydrogels were characterized using a scanning electron microscope (SEM, Hitachi, Regulus 8100) to investigate the morphology of hydrogels. Thermal properties of obtained xerogels were studied using the differential scanning calorimeter (DSC 3500 Sirius, NETZSCH, Germany)). Each xerogel sample (about 6 mg) was weighed in an aluminium pan, and

then pan was sealed and put in DSC cell. DSC measurements were performed under a nitrogen atmosphere in temperature regime from 20 to 300°C, at a heating rate of 10°C/min.

Supplementary Figures

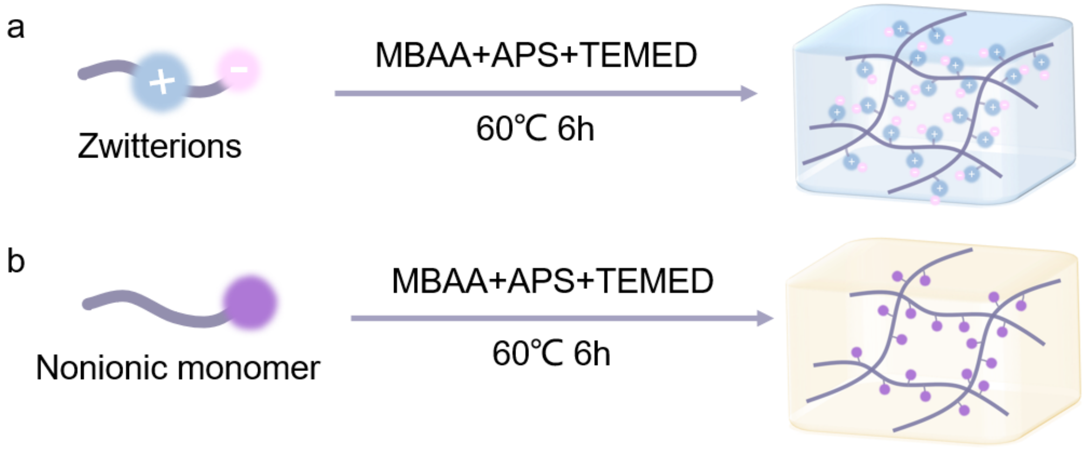

Supplementary Figure 1. The synthesis pathway of **a**, Zwitterionic and **b**, nonionic hydrogels.

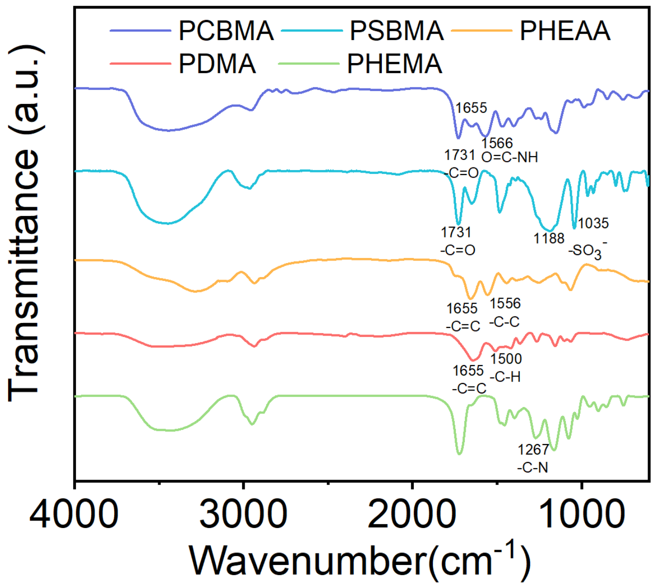

Supplementary Figure 2. FT-IR results of PCBMA, PSBMA, PHEAA, PDMA, and PHEMA hydrogels.

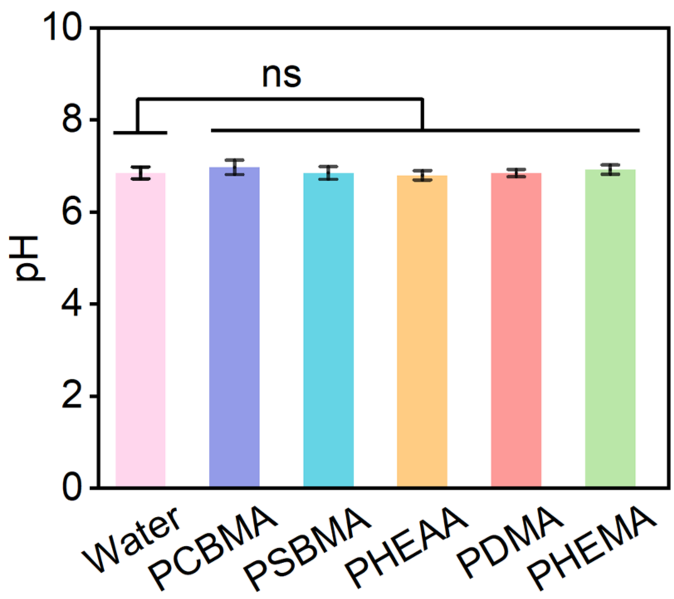

Supplementary Figure 3. The original pH value of the water phase at equilibrium balance with hydrogels. Data are reported as their means ± SDs ($N \geq 3$). ns: no significance.

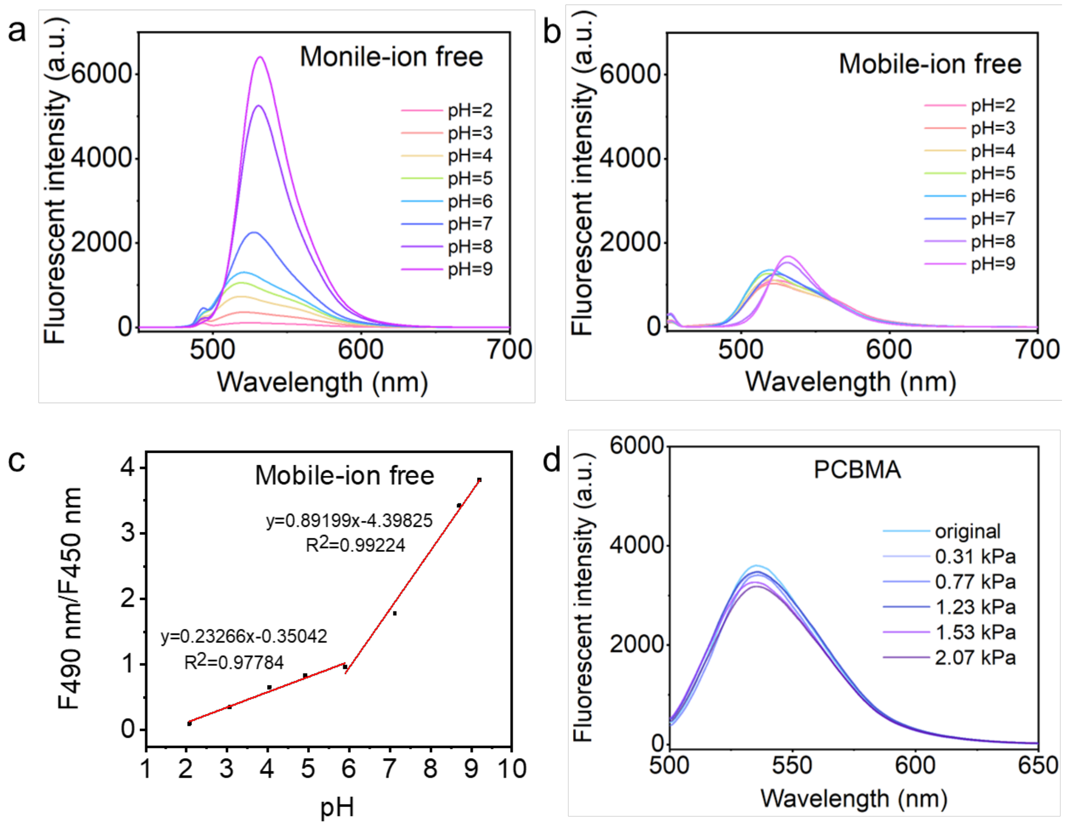

Supplementary Figure 4. a-b, Fluorescence emission spectrum of BCECF in a mobile ion-free system. Fluorescent emission spectra were recorded for excitation **a**, at 490 nm and **b**, 450 nm. **c**, The standard curve of BCECF in pure water. **d**, Fluorescence emission spectrum of the PCBMA hydrogel. Fluorescent emission spectra were recorded for excitation at 450 nm.

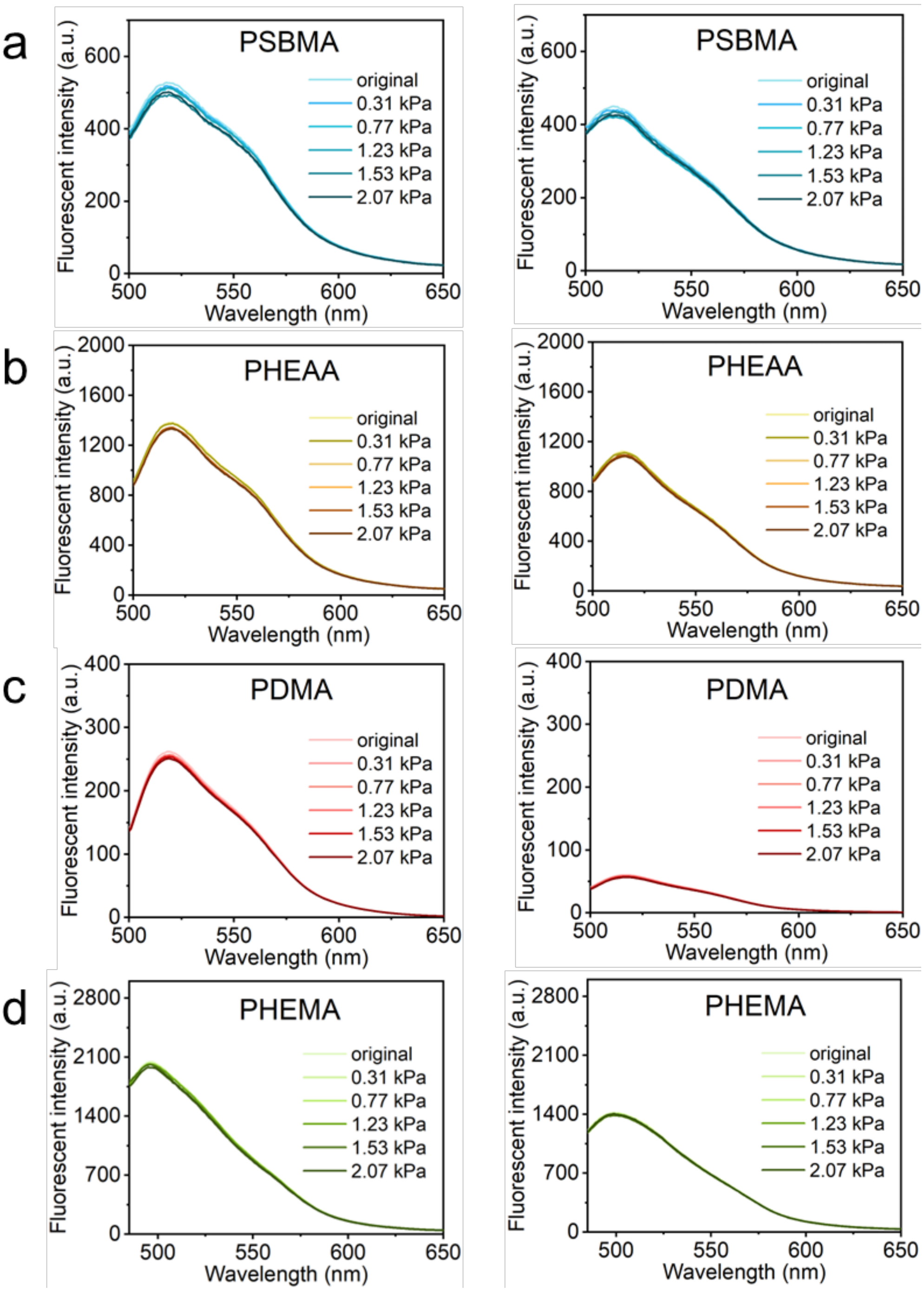

Supplementary Figure 5. Fluorescence emission spectrums. **a**, PSBMA hydrogel. **b**, PHEAA hydrogel. **c**, PDMA hydrogel. **d**, PHEMA hydrogel. Fluorescence emission spectra were recorded for excitation at 490 nm (left) and 450 nm (right).

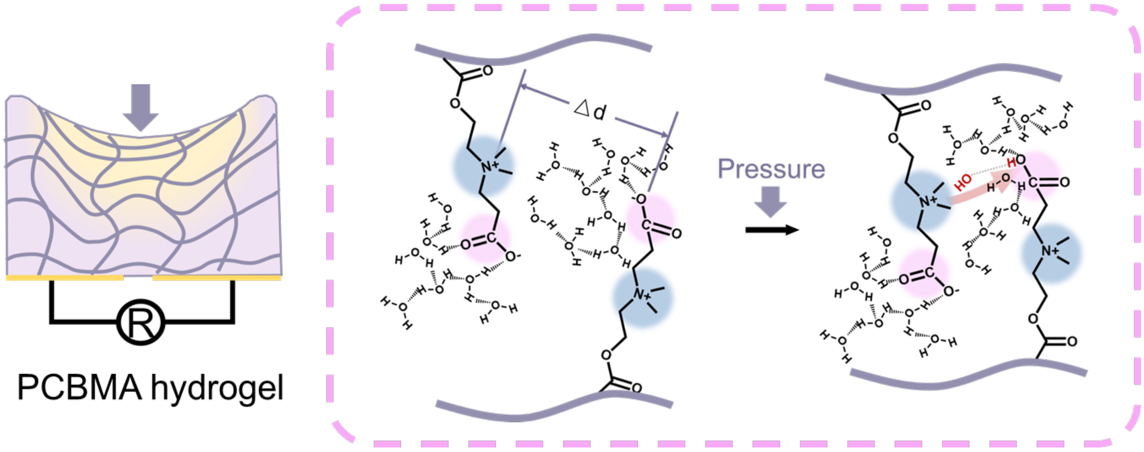

Supplementary Figure 6. Schematic diagram of the effect of intermolecular interaction on dissociation of water molecules under pressure in PCBMA hydrogel.

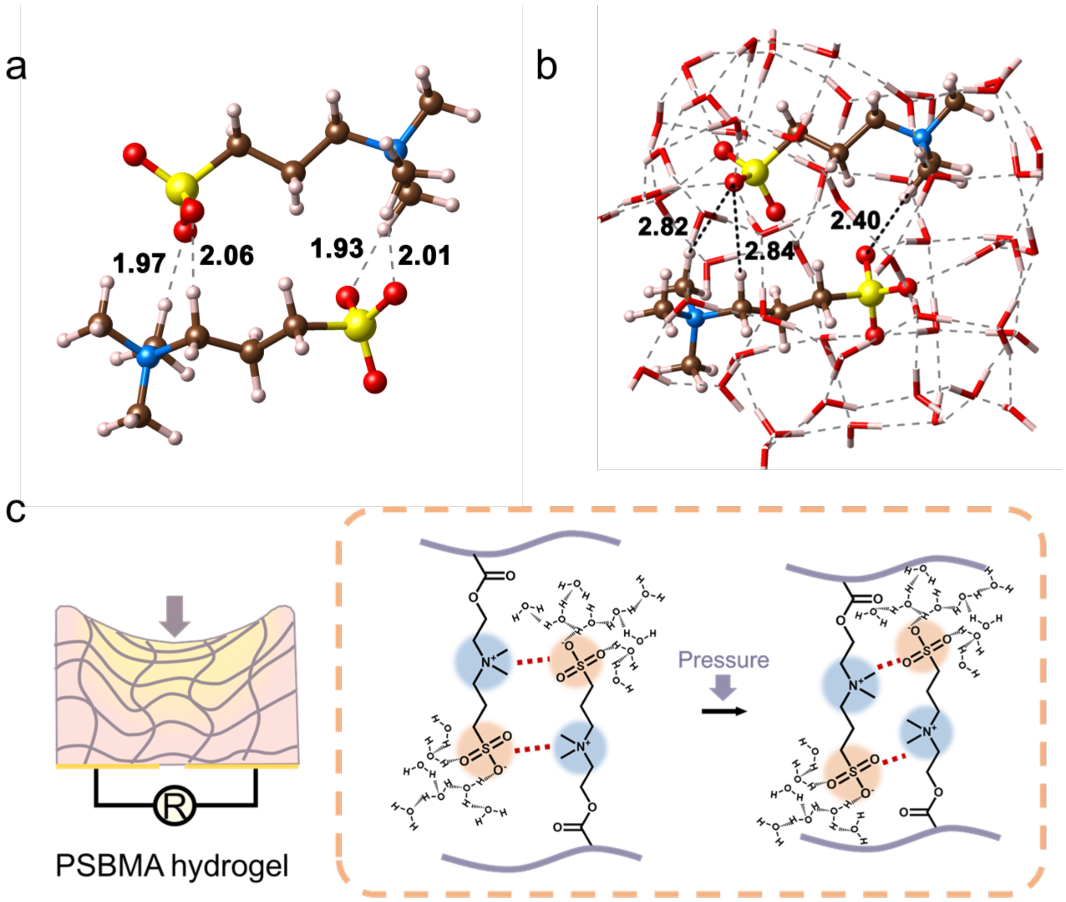

Supplementary Figure 7. DFT calculations of PSBMA hydrogel. **a**, The relaxed structure of two SBMA segments in a vacuum environment. The distances labeled in units Å corresponds to *d*, the nearest O-H distance from different molecules. **b**, The relaxed structure of two SBMA segments surrounded by 52 water molecules. (The distances labeled are in units Å). **c**, Schematic diagram of the effect of intermolecular interaction on dissociation of water molecules under pressure in PSBMA hydrogel.

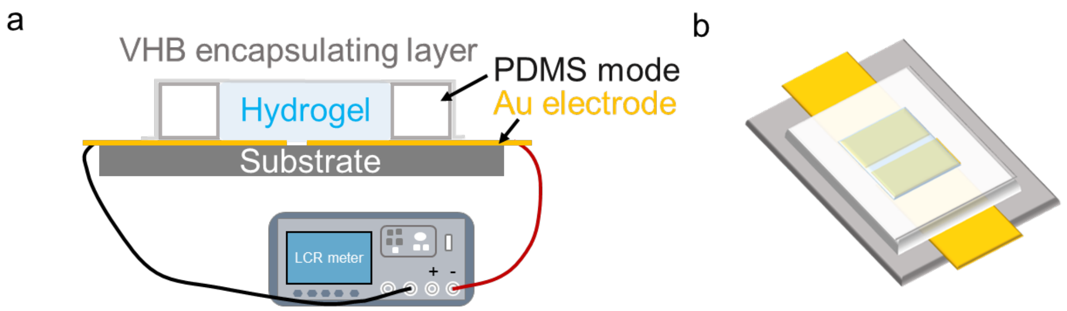

Supplementary Figure 8. Schematic diagram of the man-made device. **a**, Side view of the device. **b**, Top view of the device.

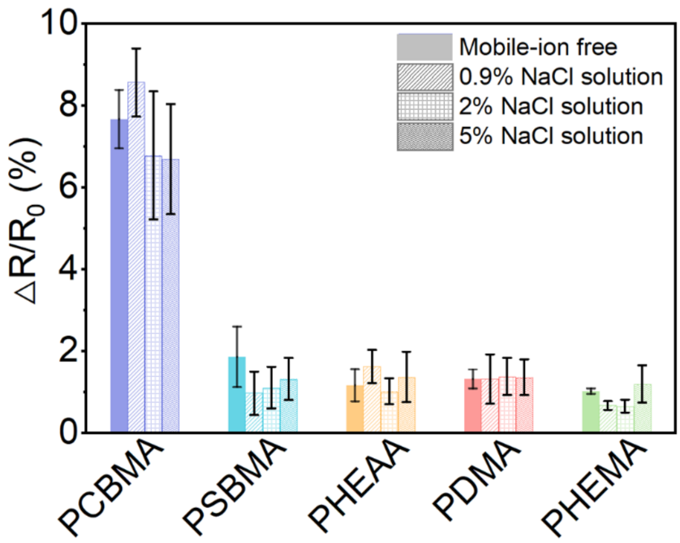

Supplementary Figure 9. The statistical graph for relative resistance variations of the hydrogels under the tiny pressure (0.77 kPa) in mobile-ion-free system and mobile-ion-addition systems. Data are reported as their means ± SDs ($N \geq 3$).

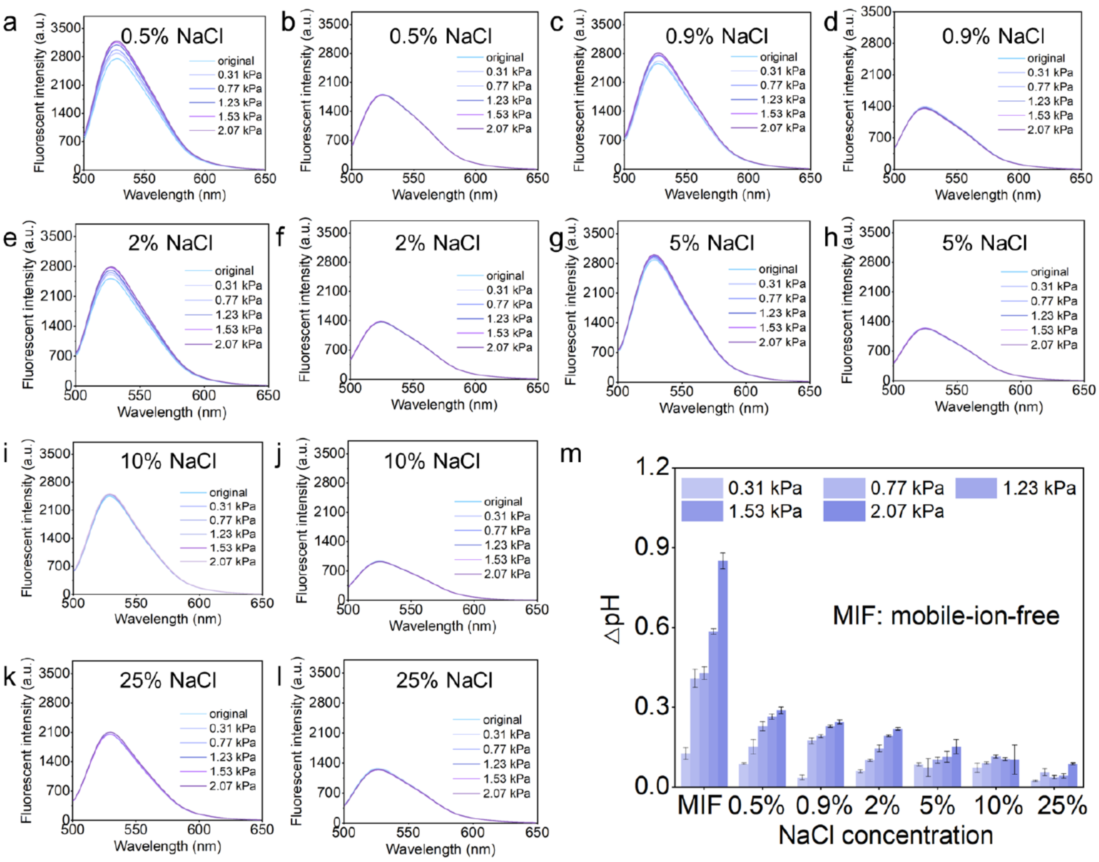

Supplementary Figure 10. a-l, Fluorescence emission spectrums of mobile-ion-added PCBMA systems with different ion concentrations. Fluorescence emission spectra were recorded for excitation at 490 nm (left) and 430 nm (right). **m**, pH changes in PCBMA hydrogels under a stress from 0.31kPa to 2.07 kPa. The size of hydrogel: 15 mm × 10 mm ×10 mm. Data are reported as their means ± SDs ($N \geq 3$). It could be observed that the fluorescence emission spectrum of PCBMA was enhanced as the stress increased from 0 to 2.07 kPa, while the enhancement was negligible in system with 10%-25% of NaCl. The pH value of PCBMA system with 0.5% of NaCl can be increased by ~0.29 under the pressure of 2.07 kPa, significantly lower than that of PCBMA in mobile-ion-free system (~0.85), while negligible response to stress in 10%-25% of NaCl system. The results indicated that the force-induced OH^- generation was inhibited by mobile-ion addition, and negligible in high-concentrated mobile ions.

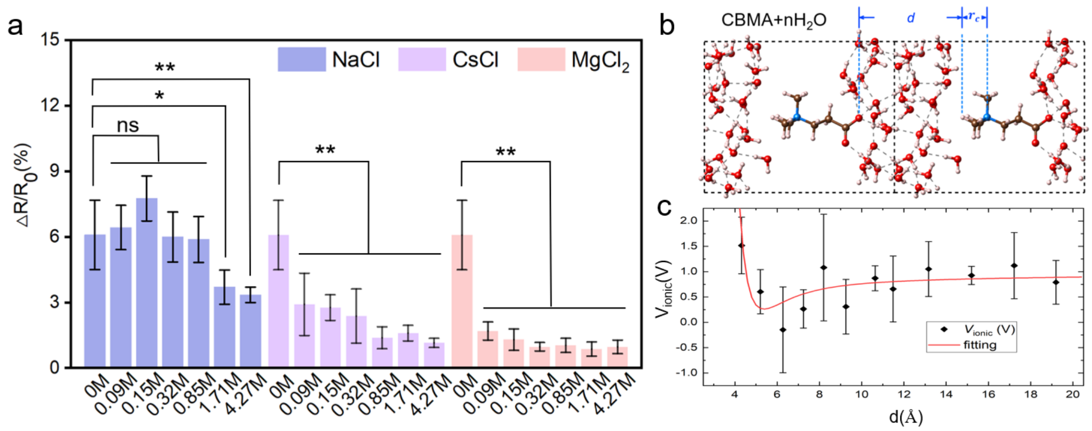

Supplementary Figure 11. a, The statistical graph for relative resistance variations of the hydrogels under the tiny pressure (0.77 kPa) in mobile-ion-free system and mobile-ion-addition systems. **b**, Graph of DFT calculations, and **c**, plots of total ionic potential versus distance *d* between $N(CH_3)^{3+}$ and COO^{-} on neighboring CBMA segments. The red solid line is fitted to Eq. (2) in the main text. Data are reported as their means ± SDs ($N \geq 3$). The sensitivity of mobile-ion-added hydrogel in figure **a** presented the different results. Under the same pressure, there was no significant reduction of R/R_0 until ion concentrations increased to 10%. The DFT calculation result shows that the monovalent cations, such as H^+, Na^+ and K^+, meets the same ionic potential energy curve with the distance between cationic and anionic groups, so they will have the similar pressure response, e.g., $CBMANa^+ + H_2O = CBMAH^+ + Na^+ + OH^-$. Therefore, when the mobile-ion addition in the hydrogel system, there would be more elements in the equilibrium influenced by the external stress. As the low-concentrated Na^+ in systems, force-induced ions (OH^- and Na^+) could be generated or increased to ensure the pressure-response sensitivity, while this phenomenon would be negligible as the high-concentrated Na^+ addition (forward tendency is completely inhibited in equilibrium, and the inhibition is proportional to the mass and charge amount of added cations).

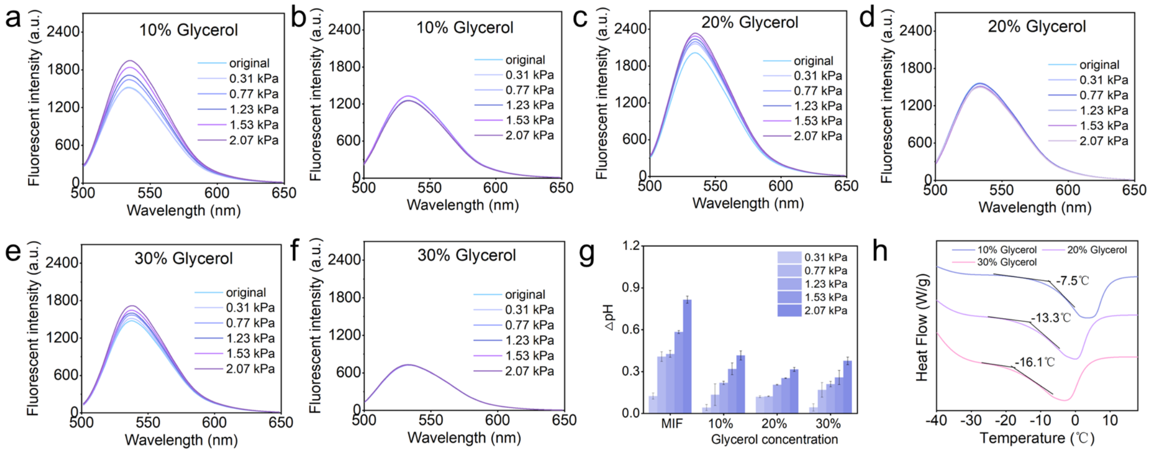

Supplementary Figure 12. a-f, Fluorescence emission spectrums of cryoprotectant-based PCBMA hydrogels with different cryoprotectant concentrations (10%, 20%, and 30%). Fluorescence emission spectra were recorded for excitation at 490 nm (left) and 430 nm (right). **g**, pH changes in hydrogels under a stress from 0.31kPa to 2.07 kPa. The size of hydrogel: 15 mm × 10 mm ×10 mm. Data are reported as their means ± SDs (N = 3). **h**, The DSC curves of hydrogels. Data are reported as their means ± SDs (*N* ≥ 3). Because zwitterionic PCBMA hydrogels have been widely reported to possess a strong hydration via ionic solvation effects[1-4], which is considered to be key for force-induced ion generation in PCBMA discovered in this work. The strong interaction between zwitterionic segments and water molecules is the necessity of equilibrium reaction ($CBMA+H_2O = CBMAH^{+}+OH^{-}$) in PCBMA hydrogel, resulting in its force-induced ion generation and sensitive pressure response. As shown in this figure, with the addition of organic cryoprotectant (glycerol) into PCBMA hydrogel, the force-induced generated OH^{-} were inhibited, resulting in slightly decreased sensitivity of hydrogel system. However, owing to the unique force-induce ion generation mechanism, the glycerol-based PCBMA hydrogel presents still satisfactory sensitivity,

as well as presenting the anti-freezing performance. This zwitterionic hydrogel would open a new opportunity for the mobile-ion-free hydrogel-based sensor (such as anti-freezing skin sensor[5-9]) due to the drawbacks of hydrogels whose conductivity depends on inorganic salt addition: ion leakage-induced conductive instability and electrode corrosion.

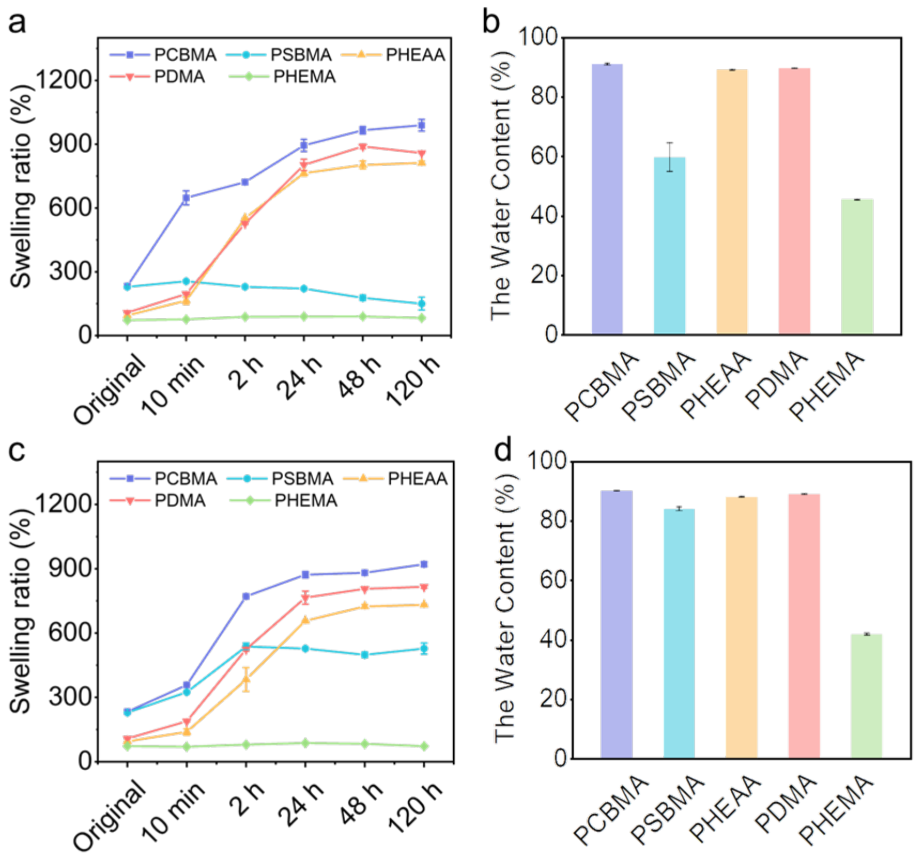

Supplementary Figure 13. a, Swelling ratios of zwitterionic and nonionic hydrogels in a mobile-ion-free system. **b**, The water contents of zwitterionic and nonionic hydrogels in a mobile ion-free system. **c**, Swelling ratios of zwitterionic and nonionic hydrogels in a mobile ion-addition system. **d**, The water contents of zwitterionic and nonionic hydrogels in a mobile ion-addition system. Data are reported as their means ± SDs ($N \geq 3$).

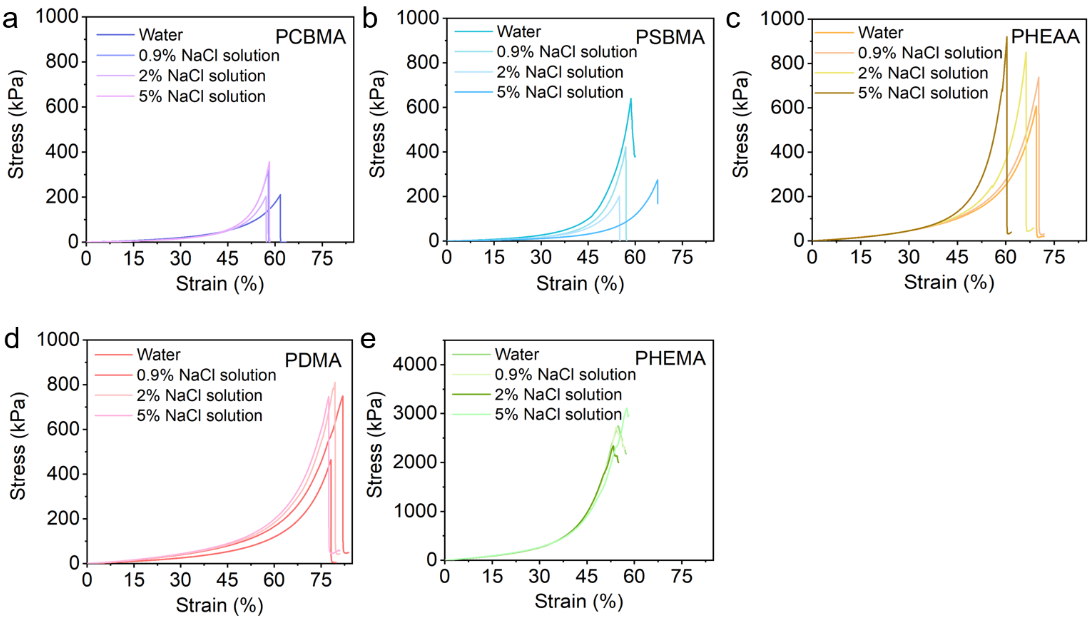

Supplementary Figure 14. Stress-strain curves of zwitterionic and nonionic hydrogels with different NaCl content during compression. **a,** PCBMA hydrogel. **b,** PSBMA hydrogel. **c,** PHEAA hydrogel. **d,** PDMA hydrogel. **e,** PHEMA hydrogel.

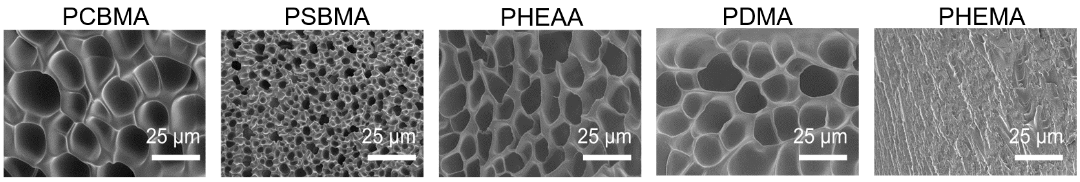

Supplementary Figure 15. The SEM images of PCBMA, PSBMA, PHEAA, PDMA, and PHEMA hydrogels (from left to right).

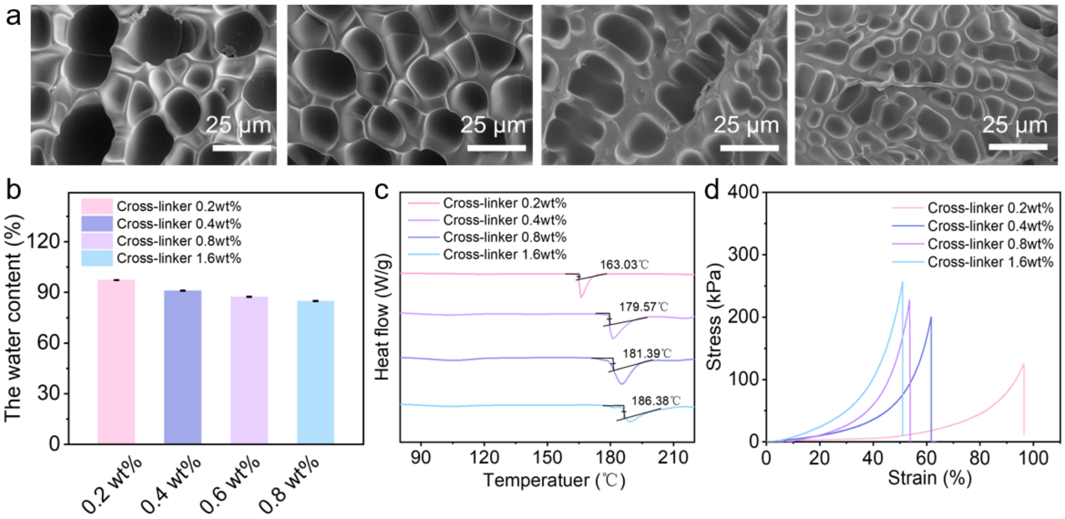

Supplementary Figure 16. a, The SEM images of PCBMA hydrogels with different crosslinking degree (the cross-linker contents are 0.2 wt%, 0.4 wt%, 0.8 wt%, and 1.6 wt%). **b**, The water content. Data are reported as their means ± SDs ($N \geq 3$). **c**, The DSC curves. **d**, The stress–strain curves.

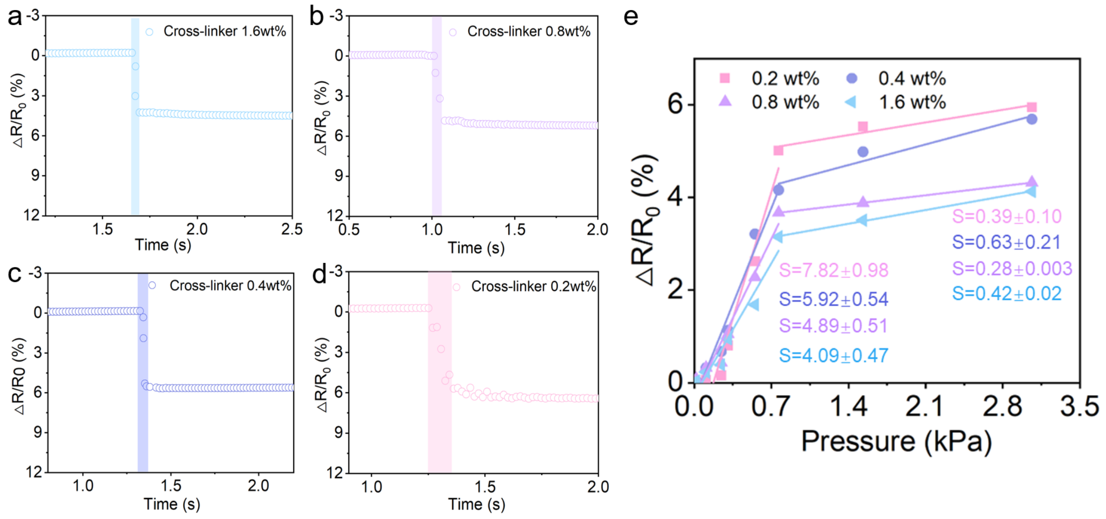

Supplementary Figure 17. a-d, The response time of PCBMA hydrogels with different crosslinking degrees during compression (the cross-linker contents are 0.2 wt%, 0.4 wt%, 0.8 wt%, and 1.6 wt%, from **a** to **d**). **e**, Comparison of the sensitivities among the PCBMA hydrogels with different crosslinking degrees.

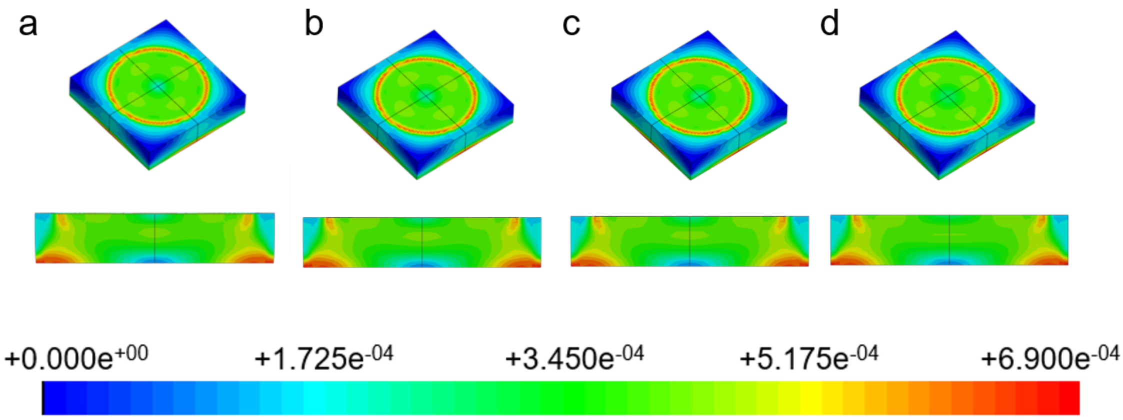

Supplementary Figure 18. Stress distribution of simulation results for PCBMA hydrogels with different crosslinking degrees under tiny pressure (0.77 kPa) (the cross-linker contents are 0.2 wt%, 0.4 wt%, 0.8 wt%, and 1.6 wt%, from **a** to **d**). The modulus of hydrogels is assigned according to the data in Supplementary Fig. 16d. The colors of the hydrogels indicate the total stress distribution.

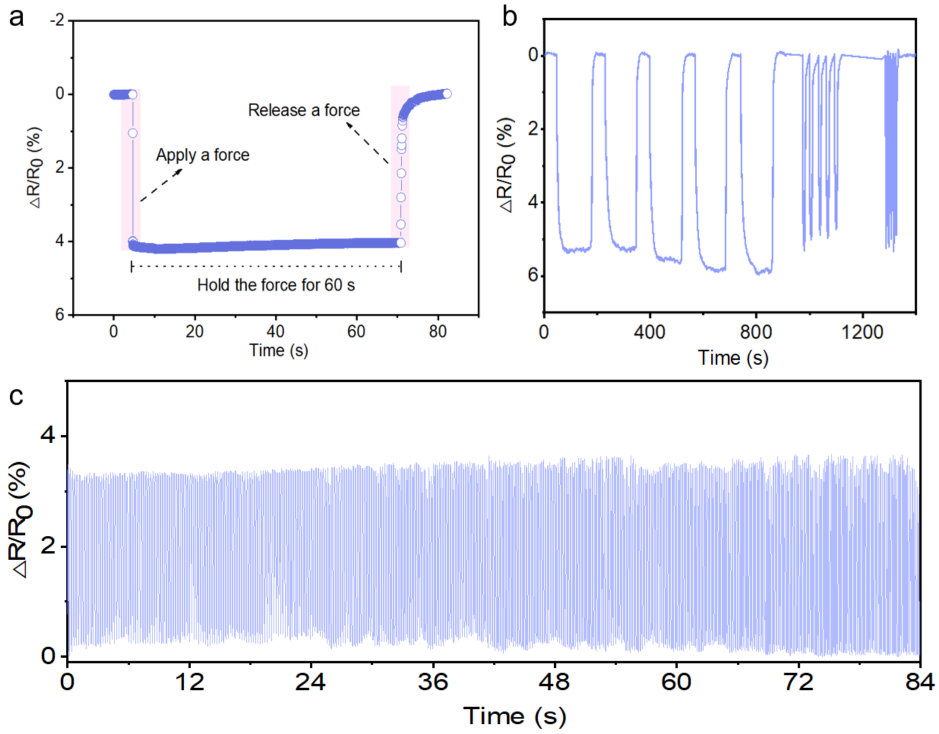

Supplementary Figure 19. **a**, Relative resistance variation under the static force. **b**, Resistance signal variation of the PCBMA hydrogel applied a dynamic force on the device. **c**, Reliability tests of the PCBMA hydrogel by loading and unloading a force on the device for 1000 cycles.

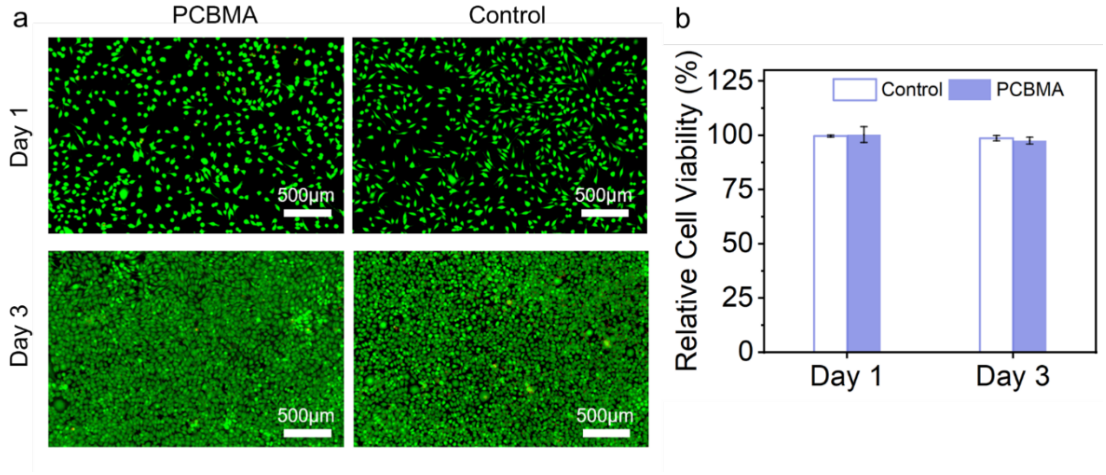

Supplementary Figure 20. a, Fluorescence images of live/dead-stained 3T3 cells cultured in the PCBMA hydrogel extract for 1 day and 3 days with comparisons against cells cultured in medium. **b**, Cell survival efficiency in control culture medium and PCBMA hydrogel extract. Data are reported as their means ± SDs ($N \geq 3$).

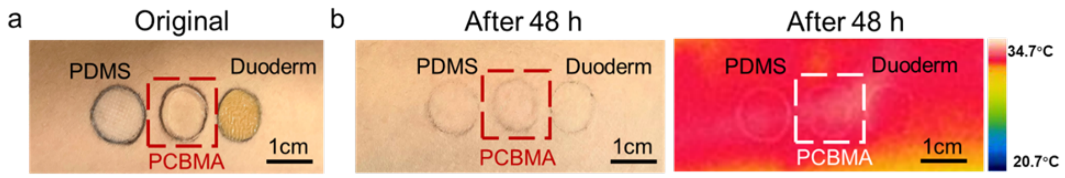

Supplementary Figure 21. Standard and IR images of the PCBMA hydrogel compared with PDMS and Durderm attached to the human skin **a,** at original state and **b,** after 48 h.

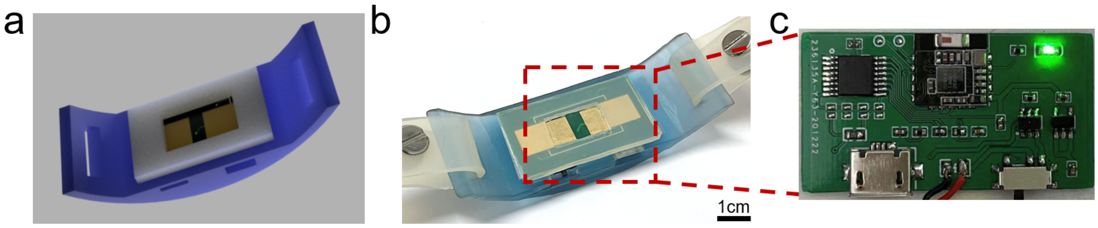

Supplementary Figure 22. a, Schematic illustration and **b**, photograph of the throat-worn sensor. **c**, Photograph of W-PCB.

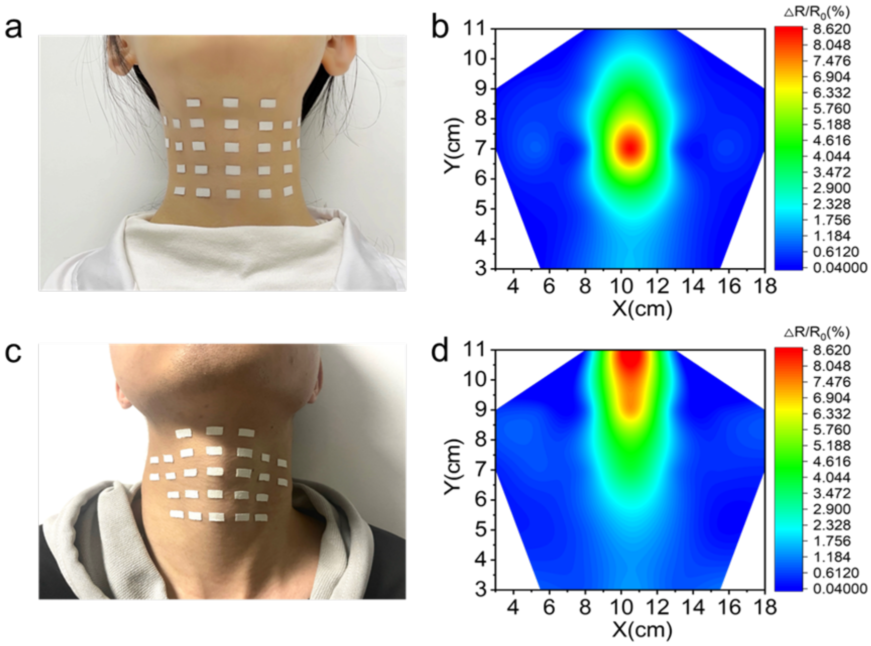

Supplementary Figure 23. a, Photograph of a female throat with 27 spatial locations. **b**, The resistance signal mapping during the same throat vibration. **c**, Photograph of a male throat with 27 spatial locations. **d**, Resistance signal mapping during the same throat vibration.

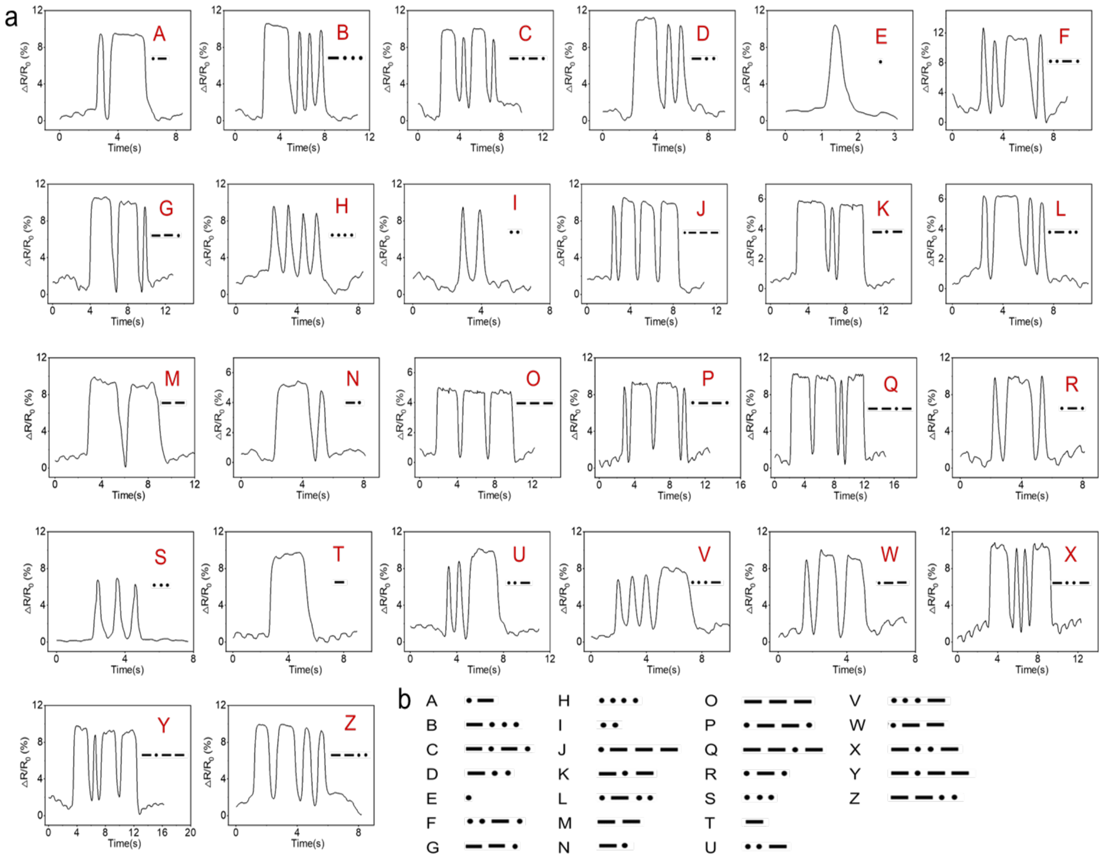

Supplementary Figure 24. a, Recognition of 26 instructions. **b**, Morse code alphabet.

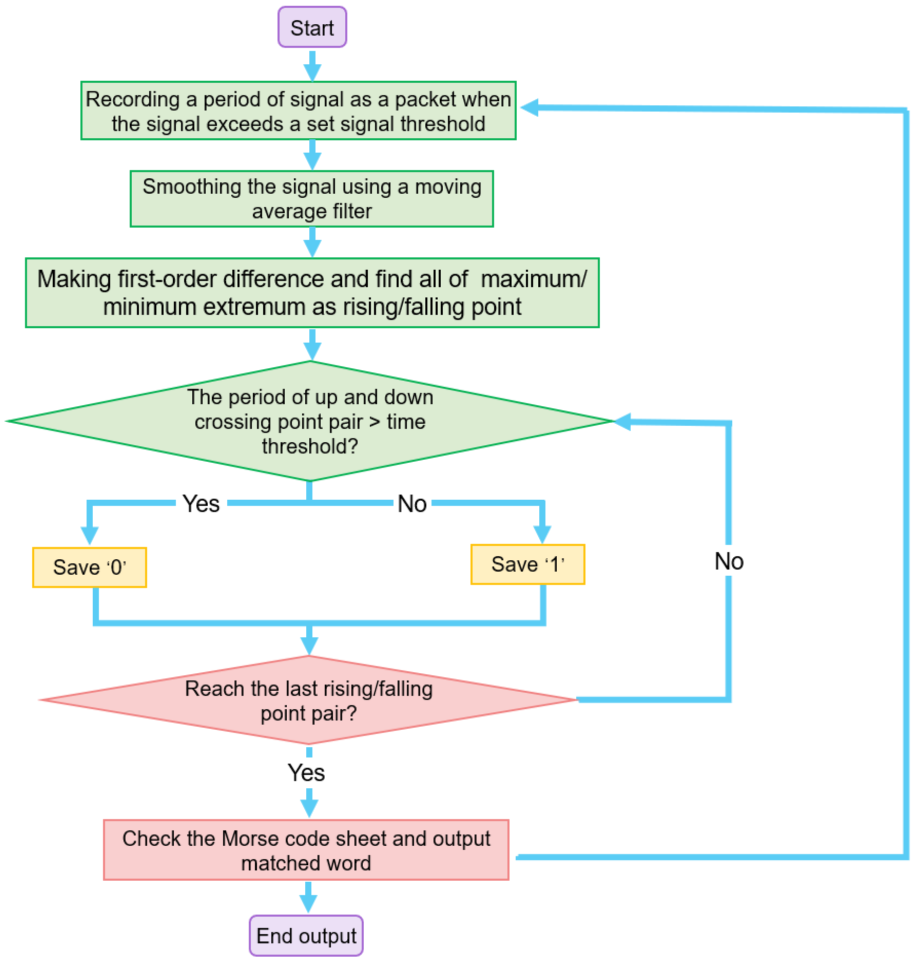

Supplementary Figure 25. Signal translation flow chart of TW-SSRS.

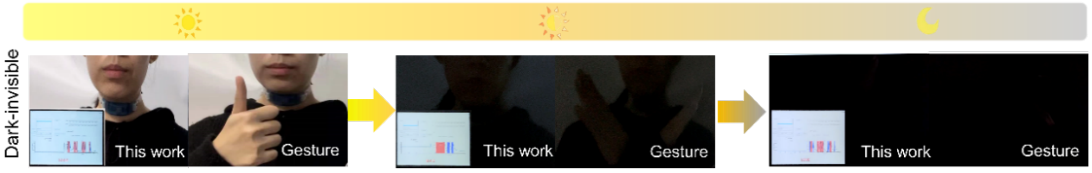

Supplementary Figure 26. Adaptability in the dark environment. The comparison of recognition effect of throat vibration (left) and Sign Language (right) in the dark environment.

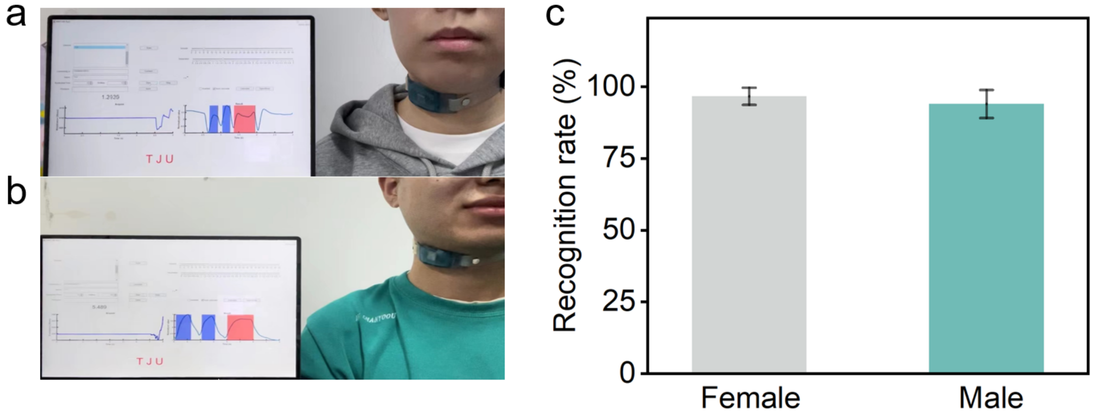

Supplementary Figure 27. The movement of detection sites on the throats of volunteers by this sensor. **a,** Photograph of a female volunteer. **b,** Photograph of a male volunteer. **c,** The recognition results of this system. The results show the average recognition rates of five instructions are 96.6% (female) and 94.0% (male) (each instruction is repeated for 30 times). No significant decrease of recognition rates is presented. Data are reported as their means ± SDs ($N \geq 3$).

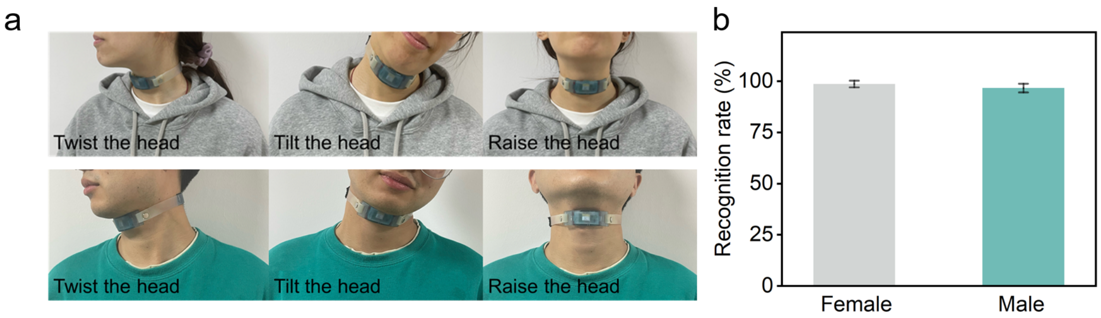

Supplementary Figure 28. The recognition of instructions when the volunteers performed a series of head movements. **a,** Photographs of a female volunteer and a male volunteer. **b,** The recognition results of this system. The results show the average recognition rates of five instructions are 98.6% (female) and 96.6% (male) (each instruction is repeated for 30 times). No significant decrease of recognition rates is presented. Data are reported as their means ± SDs ($N \geq 3$).

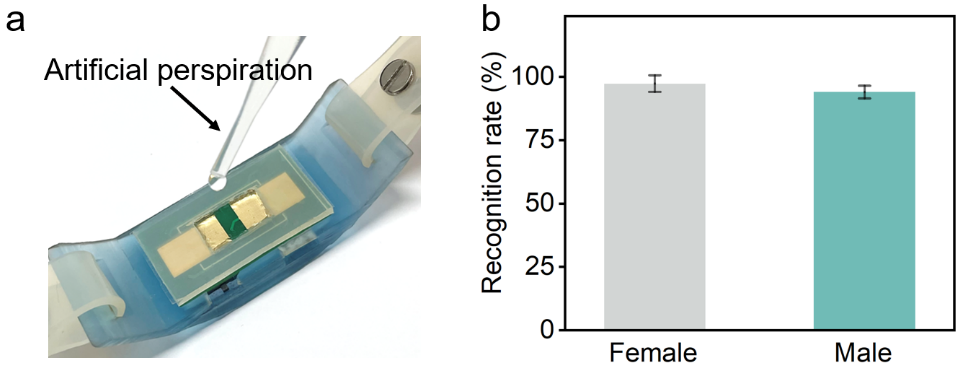

Supplementary Figure 29. The sweat-interference test on the sensor. **a,** Schematic diagram of this PCBMA-based sensor. **b,** The recognition results of the system. The results show the average recognition rates of five instructions are 97.3% (female) and 94.0% (male) (each instruction is repeated for 30 times). No significant decrease of recognition rates is presented. Data are reported as their means ± SDs ($N \geq 3$).

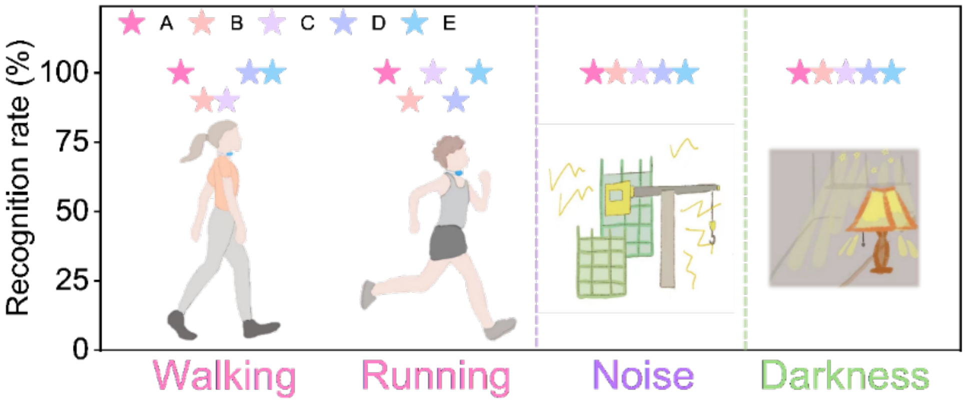

Supplementary Figure 30. The recognition rates of five instructions during walking and running, under noise and dark environment, respectively. (Each instruction is repeated for ten times).

Supplementary Tables

Supplementary Table 1. A series of different crosslinking degree of hydrogel samples.

Hydrogel	CBMA	MBAA	APS	TEMED	Water
1	2.1 g	0.0125 g	0.01 g	10 μL	6 mL
2	2.1 g	0.025 g	0.01 g	10 μL	6 mL
3	2.1 g	0.05 g	0.01 g	10 μL	6 mL
4	2.1 g	0.1 g	0.01 g	10 μL	6 mL

Supplementary Notes

Supplementary Note 1. Density functional theory (DFT) calculation details of the mobile-ion-free hydrogel systems.

DFT calculations were performed with projector augmented wave pseudopotentials[10,11], implemented in the Vienna ab initio simulation package (VASP)[12]. The generalized gradient approximation (GGA) in the Perdew, Burke, and Ernzerhof (PBE) formation was used as the exchange-correlation energy[13]. An energy cutoff of 600 eV is used for the plane-wave expansion throughout the calculations. The DFT-D3 method[14] with inclusion of van der Waals correction was employed. The periodic unit cell containing one CBMA molecule has the lattice constant of a = b = 12.0 Å and c = 12.0~24.0 Å with various number of water molecules. The position of the nitrogen atom is fixed, and other atoms were optimized until the atomic forces on each atom were less than 0.01 eV/Å.

Corresponding to equation 2:

$$\Delta E(d)=\Delta E_o+\varepsilon\left[\left(\frac{r_0+r_c}{d+r_c}\right)^{12}-\left(\frac{r_0+r_c}{d+r_c}\right)^{6}\right]-\alpha k_e\frac{e^2}{d+r_c}$$

where ΔE_0 is $\Delta E\ (d)$ at infinite distance, ε is the coefficient of the L–J 12-6 energy, r_0 corresponds to the distance for zero potential energy of the L–J 12-6 energy, r_c is the size parameter of the cationic $N(CH_3)^{3+}$ groups, α is the effective screening parameter of the Coulomb potential, k_e is the Coulomb constant, and e is the elementary charge. $\Delta E_0 = 11.4\,eV$ (263 kcal/mol), $\varepsilon = 1.6\,eV$ (37 kcal/mol), $r_0 = 4.2\,Å$, $r_c = 1.9Å$, $\alpha = 0.16$. From the above data, ΔE increases about 0.66 eV (15 kcal/mol) with the distance d changing from 5.0Å to infinity. As the positive/negative groups approach each other from infinity to 5.0Å by external forces such as pressure, the

decreasing ΔE makes the creation of OH^- anions or annihilation of H^+ cations easier and consequently increases the pH value.

Supplementary Note 2. Density functional theory (DFT) calculation details of the mobile-ion-added hydrogel systems.

The total ionic potentials of CBMA with water environments are examined by density functional theory (DFT). With various d, the nearest distance between H atom of cationic $N(CH_3)^{3+}$ group and O atom of anionic COO^- group on neighbor CBMA segments, Supplementary Fig. 11b showed the average potentials of 0.8Å~2.2Å from the O atom of anionic COO^- group to H atom of cationic $N(CH_3)^{3+}$ group. Such distance range covers most bond lengths of O-A with cationic A. The error bars correspond to the standard deviations for each case. We can find the potential follows the fitting curve in terms of eq:

$$\Delta E(d)=\Delta E_0+\varepsilon\left[\left(\frac{r_0+r_c}{d+r_c}\right)^{12}-\left(\frac{r_0+r_c}{d+r_c}\right)^{6}\right]-\alpha k_e\frac{e^2}{d+r_c}$$

with just a translation along x and y axis in the diagram. It indicated that monovalent cations., such as H^+, Na^+ and K^+, meets the same ionic potential energy curve with various distances between cationic and anionic groups, so that they will have the similar pressure response.

Supplementary Note 3. Sensitivities of zwitterionic hydrogels and nonionic hydrogels.

Sensitivity ($S=\frac{\Delta R}{R_0\cdot\Delta P}$) has been used to represent the sensing performance of pressure sensor. For zwitterionic hydrogels, whose sensitivity is directly related to the ion generation process under external pressure. Corresponding to ionic conductivity (σ) equations[15]: $\sigma=\frac{l}{RA}=\frac{N_c q^2 D}{V k_B T}=\frac{C N_A q^2 D}{k_B T}$ (S4), where N_c is the number of mobile ions ($N_c = C\cdot V\cdot N_A$), q is the charge of the mobile ions, D is the ionic diffusivity, k_B is the

Boltzmann constant, T is the temperature. The resistance (R) of hydrogel-based skin sensors can be given by: $R=\frac{CN_{\mathrm{A}}\mathrm{e}^2D\mathrm{A}}{k_B Tl}$. R is proportional to C. Therefore, the sensitivity is represented by $\mathrm{S}=\frac{\Delta C}{C_0\cdot\Delta P}$, where ΔC is the increase of ionic concentration induced by water dissociation, C_0 is the initial ion concentration in pure water system. Under the adiabatic approximation, the interactions inside the zwitterionic hydrogels can be described by using a sum of the Lennard-Jones 12–6 potential, Coulomb potential, the dissociation energy of water, and the binding energy between H^+ and anionic groups. The initial and final total energy E_0 and E_p of zwitterionic hydrogels can be represented respectively by:

$$\mathrm{E}_0\text{=-}N\alpha k_e\frac{\mathrm{e}^2}{d_0+r_\mathrm{c}}+N\varepsilon\left[\left(\frac{r_0+r_\mathrm{c}}{d_0+r_\mathrm{c}}\right)^{12}-\left(\frac{r_0+r_\mathrm{c}}{d_0+\mathrm{r}_\mathrm{c}}\right)^{6}\right]+n_0K_\mathrm{d}\text{-}n_0K_\mathrm{a},\ \text{(S5)}$$

$$\mathrm{E}_p\text{=-}N\alpha k_e\frac{\mathrm{e}^2}{d+r_\mathrm{c}}+N\varepsilon\left[\left(\frac{r_0+r_\mathrm{c}}{d+r_\mathrm{c}}\right)^{12}-\left(\frac{r_0+r_\mathrm{c}}{d+r_\mathrm{c}}\right)^{6}\right]+nK_\mathrm{d}\text{-}nK_\mathrm{a},\ \text{(S6)}$$

where N is the volume-unit average number of zwitterionic segments, n_0 is the average number of ions produced by dissociation of water itself, n is the average number of ions produced by water dissociation under external pressure, K_d is the dissociation constant of a single water molecule, K_a is the binding constant between H^+ and anionic groups. In hydrogels, the average ionic number could be represented by average ionic concentration: $n=C\cdot V\cdot N_\mathrm{A}$, where C is the average ionic concentration, V is the volume of hydrogels, N_A is the Avogadro constant. Based on this, C_0 and C can be derived from equations S5 and S6, respectively:

$$C_0=\frac{\mathrm{E}_0+N\alpha k_e\frac{\mathrm{e}^2}{d_0+r_\mathrm{c}}\text{-}N\varepsilon\left[\left(\frac{r_0+r_\mathrm{c}}{d_0+r_\mathrm{c}}\right)^{12}\text{-}\left(\frac{r_0+r_\mathrm{c}}{d_0+r_\mathrm{c}}\right)^{6}\right]}{(K_\mathrm{d}\text{-}K_\mathrm{a})V\cdot N_\mathrm{A}},\ \text{(S7)}$$

$$C=\frac{\mathrm{E}_p+N\alpha k_e\frac{\mathrm{e}^2}{d_0+r_\mathrm{c}}\text{-}N\varepsilon\left[\left(\frac{r_0+r_\mathrm{c}}{d+r_\mathrm{c}}\right)^{12}\text{-}\left(\frac{r_0+r_\mathrm{c}}{d+r_\mathrm{c}}\right)^{6}\right]}{(K_\mathrm{d}\text{-}K_\mathrm{a})V\cdot N_\mathrm{A}},\ \text{(S8)}$$

The contribution of the L–J 12–6 to $\Delta C = C\text{-}C_0$ can be omitted here since the change is much smaller than the change of the Coulomb energy. Therefore, the sensitivity of hydrogel-based skin sensors can be given by:

$$\mathrm{S} = \frac{\Delta C}{C_0 \cdot \Delta P} = \frac{\mathrm{E}_{\Delta p} + N\alpha k_e \mathrm{e}^2\left(\frac{1}{d+r_\mathrm{c}} - \frac{1}{d_0+r_\mathrm{c}}\right)}{\left(\mathrm{E}_0 + N\alpha k_e \frac{\mathrm{e}^2}{d_0+r_\mathrm{c}}\right)\cdot \Delta P}, \text{ (S9)}$$

For the PSBMA hydrogel, where d is approaching d_0, $\mathrm{E}_{\Delta p} = \frac{1}{2}\Delta P \cdot \Delta V$. The sensitivity can be given:

$$\mathrm{S} = \frac{\Delta V}{2\mathrm{E}_0 + 2N\alpha k_e \frac{\mathrm{e}^2}{d_0+r_\mathrm{c}}}, \text{ (S10)}$$

Supplementary Note 4. The usage method of the TW-SSRS.

The TW-SSRS need to be placed on the accurate position of user's laryngeal region (Fig. 5d, e). The users keep the mouth closed, using the tip of the tongue against the teeth, arching the tongue, and controlling the movement of the larynx by the movement of the hyoid bone and the external laryngeal muscle connected to the hyoid bone.

Supplementary References

1 Shao, Q. et al. Differences in cationic and anionic charge densities dictate zwitterionic associations and stimuli responses. *J. Phys. Chem. B* **118**, 6956-6962 (2014).

2 Shao, Q. & Jiang, S. Y. Influence of charged groups on the properties of zwitterionic moieties: A molecular simulation study. *J Phys Chem B* **118**, 7630-7637 (2014).

3 Leng, C. et al. In situ probing of the surface hydration of zwitterionic polymer brushes: Structural and environmental effects. *J. Phys. Chem. C* **118**, 15840-15845 (2014).

4 Shao, Q., He, Y., White, A. D. & Jiang, S. Y. Difference in hydration between carboxybetaine and sulfobetaine. *J. Phys. Chem. B* **114**, 16625–16631 (2010).

5 He, Z. et al. Bioinspired multifunctional anti-icing hydrogel. *Matter* **2**, 723-734 (2020).

6 Jian, Y. et al. Antifreezing and stretchable organohydrogels as soft actuators. *Research* **2019**, 2384347 (2019).

7 Morelle, X. P. et al. Highly stretchable and tough hydrogels below water freezing temperature. *Adv. Mater.* **30**, e1801541 (2018).

8 Huang, S. et al. Antifreezing hydrogel electrolyte with ternary hydrogen bonding for high-performance zinc-ion batteries. *Adv. Mater.* **34**, e2110140 (2022).

9 Jian, Y. et al. Biomimetic anti-freezing polymeric hydrogels: keeping soft-wet materials active in cold environments. *Mater. Horiz.* **8**, 351-369 (2021).

10 Blochl, P. E. Projector augmented-wave method. *Phys. Rev. B* **50**, 17953-17979 (1994).

11 Kresse, G. & Joubert, D. From ultrasoft pseudopotentials to the projector augmented-wave method. *Phys. Rev. B* **59**, 1758-1775 (1998).

12 Kresse, G. & Furthmu¨ller, J. Efficient iterative schemes for ab initio total-energy calculations using a plane-wave basis set. *Phys. Rev. B* **54**, 11169-11186 (1996).

13 Perdew, J. P., Burke, K. & Ernzerhof, M. Generalized gradient approximation made simple. *Phys. Rev. Lett.* **77**, 3865-3868 (1996).

14 Grimme, S., Antony, J., Ehrlich, S. & Krieg, H. A consistent and accurate ab initio parametrization of density functional dispersion correction (DFT-D) for the 94 elements H-Pu. *J. Chem. Phys.* **132**, 154104 (2010).

15 He, X., Zhu, Y., Epstein, A. & Mo, Y. Statistical variances of diffusional properties from ab initio molecular dynamics simulations. *NPJ Comput. Mater.* **18**, (2018).
