# Supplementary material for: Force-induced ion generation in zwitterionic hydrogels for a sensitive silent-speech sensor
Source: Nat Commun. 2023 Jan 13;14:219. doi: 10.1038/s41467-023-35893-7 (PMC9839672; doi:10.1038/s41467-023-35893-7)
Supplement: Supplementary file 2 — Description of Additional Supplementary Files [file 41467_2023_35893_MOESM2_ESM.pdf]

Description of Additional Supplementary Files

File name: Supplementary Movie 1

Description: The TW-SSRS for silent speech acquisition.

File name: Supplementary Movie 2

Description: Gender indifference. 12 volunteers (six females and six males) are given six silent instructions related to emotions (“HAPPY,” “SAD,” “SLEEPY,” “TIRED,” “CALM,” and “ANGRY”).

File name: Supplementary Movie 3

Description: Motion suitability. The recognition rates of TW-SSRS during walking (0.8 m/s) and running (1.6 m/s) are maintained as high as 92%.

File name: Supplementary Movie 4

Description: Working in noisy environment. The TW-SSRS performs well in noisy public places and quiet workplaces. The ambient noise reaches ~90 dB.

File name: Supplementary Movie 5

Description: Working in dark environment. The performance of TW-SSRS is unaffected by the visible light intensity.
